# Wavelength‐Dependent Photobiomodulation Regulates Macrophage Polarization via Mitochondrial Dynamics and Metabolic Reprogramming

**DOI:** 10.1002/advs.75920

**Published:** 2026-06-02

**Authors:** Qiusheng Shi, Hao Jia, Jianfei Dong, Linhao Li, Jingqi Cao, Xun Chen, Zhenzhen Jia, Jing Na, Zhijie Yang, Xinyuan Chen, Yubo Fan, Shuhua Yue, Lisha Zheng

**Affiliations:** ^1^ Key Laboratory of Biomechanics and Mechanobiology (Beihang University), Ministry of Education, Beijing Advanced Innovation Center for Biomedical Engineering School of Biological Science and Medical Engineering Beihang University Beijing China; ^2^ School of Future Science and Engineering Soochow University Suzhou China

**Keywords:** fatty acid oxidation, glycolysis, macrophage polarization, mitochondrial dynamics, photobiomodulation, wound healing

## Abstract

Photobiomodulation (PBM) provides a non‐invasive means to regulate immune function, yet its clinical translation is hindered by a lack of mechanistic links between light parameters and biological outcomes. Here, we demonstrate that specific wavelengths act as metabolic switches that direct macrophage polarization through the selective engagement of distinct immunometabolic pathways. In both in vitro and in vivo wound healing models, 850‐nm light enhances fatty acid oxidation and lipid droplet–mitochondria interactions, driving anti‐inflammatory M2 polarization and accelerating tissue repair. Conversely, 625 nm light increases glycolytic flux and lactate production, promoting a pro‐inflammatory M1 state that delays healing. We identify mitochondrial dynamics as the key interface: 850 and 625 nm light promote mitochondrial fusion and fission, respectively, to dictate metabolic routing. Causality was confirmed via metabolic interventions, which reversed wavelength‐specific polarization outcomes. Together, these findings define photo‐immunometabolism as a wavelength‐dependent framework in which light regulates macrophage fate through coordinated control of mitochondrial dynamics and metabolism. This framework provides a mechanistic basis for precision, wavelength‐tailored PBM therapies for wound repair and other immune‐mediated inflammatory disorders.

## Graphical abstract

Light wavelength encodes distinct macrophage fates through mitochondrial and metabolic remodeling. Red 625 nm irradiation induces glycolysis, mitochondrial fission, and M1 polarization, whereas near‐infrared 850 nm irradiation promotes fatty acid oxidation, mitochondrial fusion, and M2 polarization. This wavelength‐dependent immunometabolic switch provides a tunable strategy for directing inflammation and tissue repair.

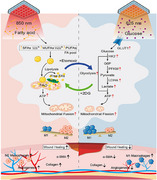

## 1 Introduction

Photobiomodulation (PBM), a widely applied non‐pharmacological therapeutic approach, utilizes low‐intensity light to modulate cellular functions and biological processes in tissues to alleviate pain and inflammation, modulate immune responses, and promote tissue healing and regeneration [1, 2]. Its non‐invasiveness, precise targeting, and excellent safety, profile have enabled PBM applications in various diseases, including dermatological conditions, diabetes, brain injury, spinal cord injury, and postoperative swelling [3]. Recently, growing dermatological research has rapidly expanded PBM use for skin wound healing, acne, scar reduction, and acute radiation dermatitis [4, 5]. However, achieving precise matching between phototherapy parameters and patient outcomes, along with the underlying molecular mechanisms of PBM in wound healing remain elusive.

Wound healing is a complex and tightly orchestrated multicellular process involving the overlapping phases of hemostasis, inflammation, proliferation, and remodeling [6]. After skin injury, exposure of the subendothelial matrix, collagen, and tissue factor activates platelet aggregation and degranulation, leading to clot formation and the release of chemokines and growth factors that provide early biochemical and structural cues for subsequent repair [7]. During inflammation, neutrophils are among the first immune cells recruited to the wound site, where they remove debris and bacteria and help establish a permissive environment for repair [8]. Monocytes/macrophages subsequently accumulate and contribute to the phagocytosis of pathogens, apoptotic cells, and damaged tissue while coordinating inflammatory and reparative signaling [9]. During proliferation, fibroblasts, keratinocytes, endothelial cells, and extracellular‐matrix components contribute to re‐epithelialization, granulation tissue formation, angiogenesis, and matrix deposition [10]. During remodeling, the provisional extracellular matrix is reorganized, immature type III collagen is gradually replaced by mature type I collagen, and tissue architecture is restored [11]. Because persistent or dysregulated inflammation compromises granulation tissue formation, angiogenesis, and matrix remodeling [12], precise modulation of the wound inflammatory microenvironment is critical for effective tissue repair.

PBM has been reported since the 1960s to alleviate back and neck pain, modulate inflammation, and improve wound‐healing outcomes [13, 14]. Wavelengths of 500–700 nm are typically suitable for superficial tissue trauma, whereas 800–1000‐nm light achieves better penetration for deeper injuries [15]. However, irradiation conditions, including wavelength, energy density, exposure duration, spot size, and illumination‐field uniformity, vary substantially across studies, leaving the relative immunoregulatory efficacy of red versus near‐infrared (NIR) light remains debated [16]. For example, 660‐nm irradiation was better than 780‐nm irradiation (at 20 mW output power, 500 mW/cm^2^ power density, 5 J/cm^2^ energy density) in reducing tissue destruction and inflammation, along with promoting collagen deposition in rats with third‐degree burns [17]. In contrast, other studies report superior NIR effects under matched dosimetry. For instance, in a mouse partial‐thickness dermal abrasion model treated at the same fluence (4 J/cm^2^) and fluence rate (10 mW/cm^2^), 810‐nm irradiation accelerated wound closure more than 635‐nm irradiation [18]. Similarly, in vitro 810‐nm irradiation has been shown to suppress pro‐inflammatory cytokine release more effectively than that at 660 nm [19]. These heterogeneous findings underscore the need for a rigorously controlled experimental framework to establish relationships between PBM parameters and inflammatory regulation during wound repair to enable mechanistic elucidation and more precise clinical translation.

Among the cellular components involved in wound repair, macrophages are particularly important immunoregulatory cells because they participate in debris clearance, inflammation resolution, angiogenesis, fibroblast activation, extracellular‐matrix remodeling, and the transition from inflammation to tissue repair [7]. Their functional plasticity enables them to adopt distinct activation states in response to local microenvironmental cues [20]. Classically activated M1 macrophages predominate during the early inflammatory phase, where they activate pro‐inflammatory signaling pathways, produce reactive oxygen and nitrogen species, and secrete cytokines such as TNF‐α and IL‐1β [21]. In contrast, alternatively activated M2 macrophages contribute to inflammation resolution and tissue remodeling by producing mediators such as IL‐10, TGF‐β, and matrix metalloproteinases [22]. An imbalanced M1/M2 macrophage ratio has been associated with persistent inflammation and impaired tissue repair [23]. Despite evidence showing that PBM can reduce inflammation, the specific effects of PBM on macrophage polarization remain poorly defined.

Macrophage polarization is closely coupled to mitochondrial state and metabolic configuration [24]. Pro‐inflammatory macrophages typically rely more heavily on glycolysis, whereas anti‐inflammatory or reparative macrophages are more dependent on oxidative phosphorylation (OXPHOS) [25]. Increasing evidence further indicates that mitochondrial dynamics are integrated with these metabolic states: mitochondrial fission is commonly associated with fragmented mitochondrial networks, glycolytic rewiring, and inflammatory activation, whereas mitochondrial fusion is generally linked to elongated mitochondrial morphology, preserved oxidative metabolism, and reparative macrophage programs [26]. Mitochondrial dynamics should therefore be viewed not merely as structural changes in organelle shape, but as part of a broader regulatory axis connecting bioenergetic state, substrate utilization, and macrophage phenotype [27]. Reportedly, mitochondria are major intracellular photoacceptors for red and near‐infrared light, and PBM has been shown to modulate mitochondrial activity and downstream signaling [28, 29]. However, whether PBM can directionally regulate macrophage polarization through mitochondrial dynamics and metabolic reprogramming remains unclear.

To address these gaps, this study aimed to define how PBM wavelength regulates macrophage polarization and cutaneous wound repair under rigorously controlled irradiation conditions. We developed a custom‐built PBM platform that delivers spatially uniform red and NIR light at 625, 850, and 935 nm with controlled irradiance and dosimetry. Using this platform, we determined whether distinct wavelengths induce divergent macrophage polarization states and whether these wavelength‐dependent immune responses are associated with differential wound‐healing outcomes in vivo. We further investigated mitochondrial dynamics and metabolic reprogramming as mechanistic links between wavelength input and macrophage fate. Together, these findings establish a wavelength‐dependent photo‐immunometabolic framework for macrophage regulation and provide a mechanistic basis for precision wavelength selection in wound‐repair therapy.

## 2 Results

### 2.1 Light Wavelength Drives Macrophage Polarization

**FIGURE 1:**
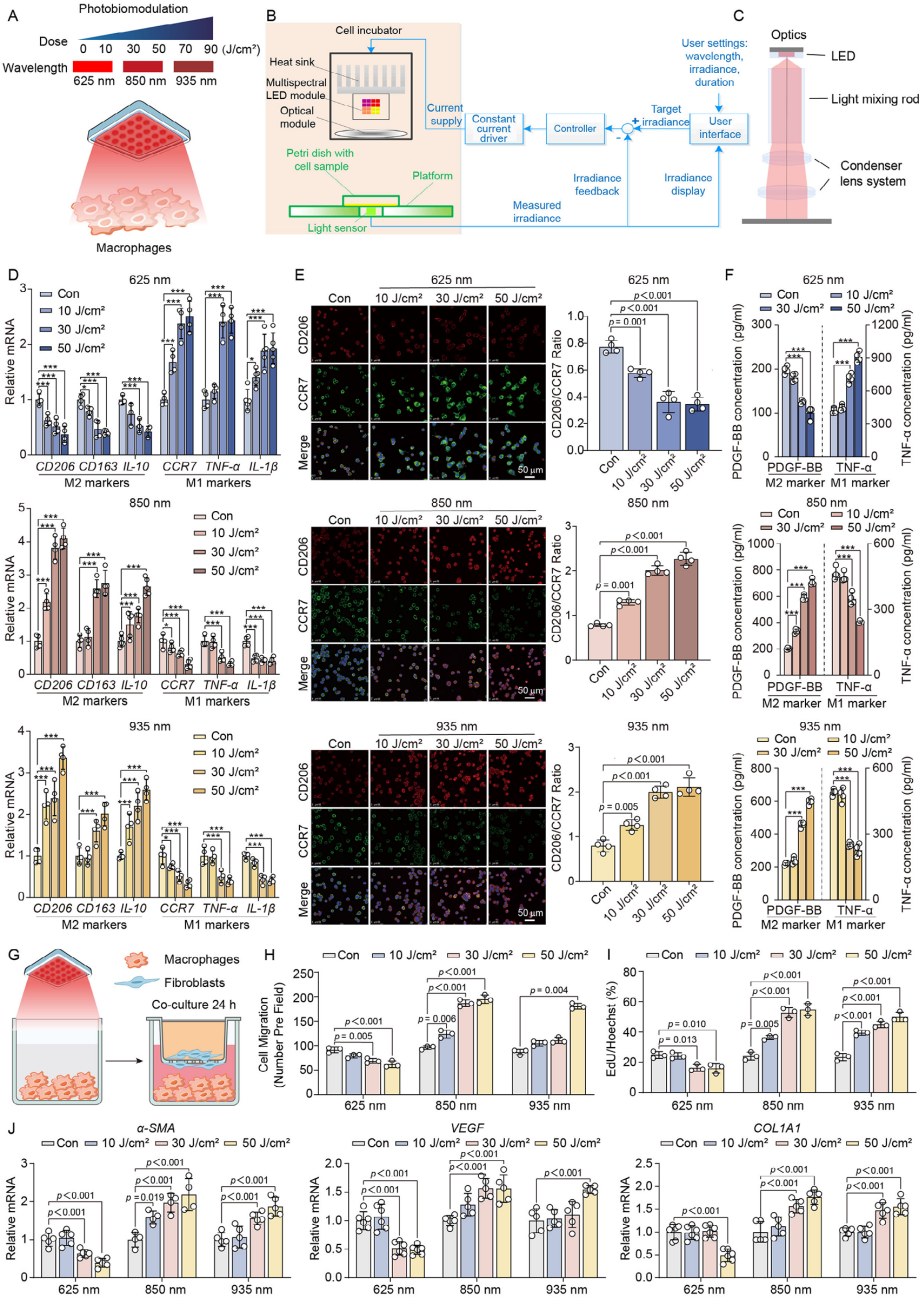
Light wavelength drives macrophage polarization and differentially regulates fibroblast functions. (A) Schematic of the custom‐built PBM setup delivering uniform red and NIR irradiation at 625, 850, and 935 nm with controlled energy density (10, 30, 50, 70, and 90 J/cm^2^), and the experimental workflow for assessing wavelength‐dependent macrophage polarization. (B) Schematic of the control architecture. The multispectral LED module was housed inside the incubator, while external electronics, including the current supply, controller, and user interface, enabled user‐defined control of wavelength, irradiance, and exposure duration. (C) Schematic of the optical module, including the LED source, light‐mixing rod, and condenser lens assembly used to homogenize and deliver illumination to the sample. (D) qRT–PCR analysis of polarization‐associated genes in THP‐1 monocyte‐derived macrophages after irradiation at 625, 850, or 935 nm within the non‐toxic dose range (10, 30, and 50 J/cm^2^): M1‐associated genes (*CCR7*, *TNF‐α*, *IL1β*) and M2‐associated genes (*CD206*, *CD163*, *IL10*) (*n* ≥ 4 independent biological experiments). (E) Representative immunofluorescence images and quantification of CD206 (red) and CCR7 (green) under the conditions in (D); the CD206/CCR7 fluorescence intensity ratio is shown (scale bar, 50 µm; *n* = 4 independent biological experiments). (F) ELISA quantification of TNF‐α and PDGF‐BB in culture supernatants 24 h after irradiation as in (D) (*n* = 4 independent biological experiments). (G) Schematic of the Transwell co‐culture system to evaluate macrophage‐mediated effects on fibroblasts after wavelength‐specific irradiation. (H) Quantification of fibroblast migration in Transwell co‐culture with macrophages pre‐exposed to 625, 850, or 935 nm irradiation (*n* = 3 independent biological experiments). (I) EdU incorporation assay assessing fibroblast proliferation in co‐culture with wavelength‐treated macrophages (*n* = 3 independent biological experiments). (J) qRT–PCR analysis of fibroblast activation/wound‐healing–related genes (*α‐SMA*, *COL1A1*, *VEGF*) after co‐culture with wavelength‐treated macrophages (*n* ≥ 4 independent biological experiments). Data are presented as mean ± s.d. Exact *p* values or significance levels are indicated in the figure. ^*^ *p* < 0.05 and ^***^ *p* < 0.01.

To investigate the PBM‐mediated regulation of macrophage phenotype, we developed a custom‐built PBM platform that delivered uniform red and NIR light at 625, 850, and 935 nm across defined energy densities of 10, 30, 50, 70, and 90 J/cm^2^ (Figure 1A). Macrophage phenotype was characterized using a panel of established polarization‐associated markers [30, 31]. At the gene‐expression level, *CCR7*, *TNF‐α*, and *IL1β* were used as M1‐associated markers, whereas *CD206*, *CD163*, and *IL10* were used as M2‐associated markers. At the imaging level, CD206/CCR7 dual‐immunofluorescence staining was used to visualize macrophage phenotype, and the CD206/CCR7 fluorescence intensity ratio was quantified to assess the relative M2/M1 shift. At the secreted‐protein level, enzyme‐linked immunosorbent assay (ELISA) quantification of TNF‐α and platelet‐derived growth factor‐BB (PDGF‐BB) provided complementary validation of inflammatory/M1‐associated and reparative/M2‐associated responses, respectively. The system comprised a multispectral LED module housed inside the incubator together with external control electronics that allowed precise adjustment of irradiation conditions while minimizing environmental fluctuation during treatment (Figure S1A). Spectral characterization with a Maya2000Pro spectrometer verified the emission profiles of the 625‐, 850‐, and 935‐nm LEDs (Figure S1B). To ensure dosimetric stability, irradiance was regulated through a closed‐loop feedback strategy and continuously monitored by an integrated photodiode (Figure 1B), maintaining output at all three wavelengths within ±5% of the target setpoint during exposure (Figure S1C). To improve field homogeneity, the optical module incorporated a light‐mixing rod and condenser lens assembly (Figure 1C), achieving >85% irradiance uniformity across a defined 5 × 5 cm illumination area at a reference intensity of 50 mW/cm^2^ (Figure S1D). Detailed dosimetry parameters for each wavelength are provided in Table S1. All in vitro assays were performed after a single irradiation exposure at the indicated wavelength and dose, rather than repeated irradiation. Cytotoxicity assays showed that doses below 50 J/cm^2^ did not affect the viability of THP‐1 monocyte‐derived macrophages, whereas 70 and 90 J/cm^2^ were cytotoxic (Figure S2). Subsequent experiments therefore focused on doses of 10, 30, and 50 J/cm^2^. Within this range, qRT‐PCR and immunofluorescence analyses were performed after completion of irradiation, whereas culture supernatants for ELISA were collected 24 h later. Increasing doses of 625‐nm red light enhance expression of M1‐related genes (*CCR7*, *TNF‐α*, and *IL1B*) while suppressing M2‐related genes (*CD206*, *CD163*, and *IL10*). In contrast, NIR light at 850 and 935 nm produced graded upregulation of M2‐related genes and downregulated M1‐related genes (Figure 1D). Immunofluorescence staining confirmed that 625‐nm red light decreased the CD206/CCR7 ratio, whereas 850‐ or 935‐nm NIR light increased this ratio (Figure 1E). Consistently, ELISA showed that 625‐nm red light increased the secretion of the pro‐inflammatory cytokine TNF‐α and reduced the level of the reparative factor PDGF‐BB, whereas 850‐ and 935‐nm NIR light produced the opposite effects (Figure 1F). Similar wavelength‐specific polarization modulation occurred in mouse bone marrow‐derived macrophages (BMDMs) (Figure S3A–C). These results aligned with classical biochemical polarization induced by lipopolysaccharide + interferon‐γ (M1) versus IL‐4 + IL‐13 (M2) (Figure S4A–D). To exclude potential PBM‐associated thermal effects, we monitored temperature changes across wavelength‐specific irradiation conditions and performed temperature‐matched control tests at 37.5°C, 38.0°C, and 38.5°C during exposure (Figure S5A,B). Notably, temperature changes alone did not significantly alter polarization markers (Figure S5C,D). Altogether, these findings demonstrate that wavelength‐specific PBM provides a non‐invasive, biochemical factor‐free approach to regulate macrophage polarization.

Macrophage phenotypic switching regulates fibroblast functions, from migration to matrix remodeling, during wound healing [12]. Herein, Transwell co‐culture assays were performed in which macrophages exposed to 625‐, 850‐, or 935‐nm light were co‐cultured with fibroblasts (Figure 1G). NIR light at 850 and 935 nm enhanced fibroblast migration, proliferation, and expression of *α‐SMA*, *COL1A1*, and *VEGF*, whereas 625‐nm red light exerted opposite effects (Figure 1H–J and Figure S6A,B). Across NIR wavelengths, 850‐nm irradiation elicited stronger effects on fibroblasts than 935‐nm irradiation (Tables S2–S4). Overall, 625‐nm red light drove macrophages toward an M1 phenotype that impaired fibroblast behavior, whereas 850‐ and 935‐nm NIR light induced an M2 phenotype and enhanced fibroblast migration and proliferation.

### 2.2 Wavelength‐Specific Effects of PBM on Wound Healing

**FIGURE 2:**
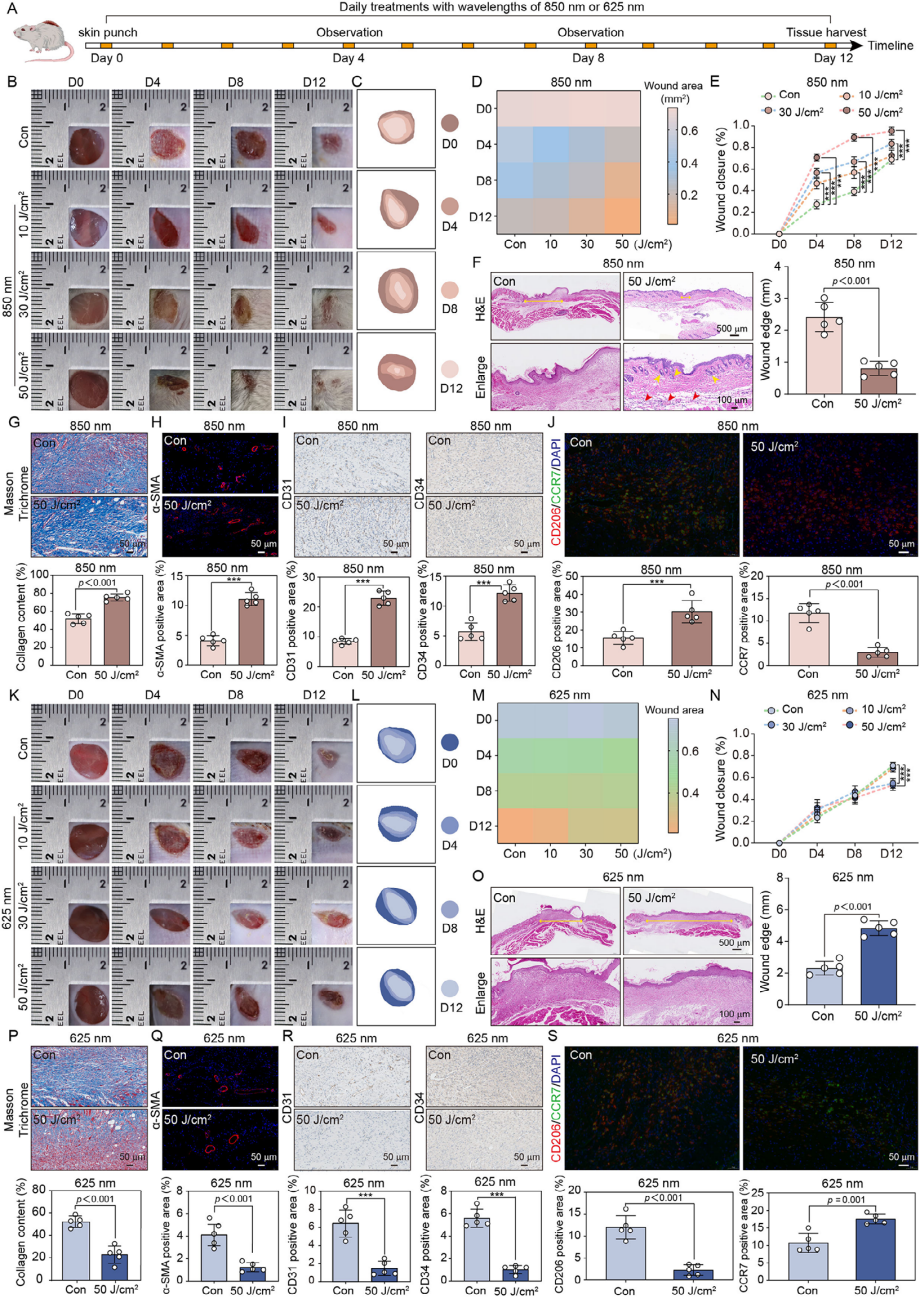
Wavelength‐specific effects of PBM on cutaneous wound healing in vivo. (A) Schematic of the full‐thickness excisional wound model in BALB/c mice, showing the experimental timeline and PBM regimen using 850‐nm NIR or 625‐nm red light at 10, 30, or 50 J/cm^2^. (B) Representative photographs of wounds on days 0, 4, 8, and 12 from mice treated with 850‐nm PBM at the indicated doses. (C) Traced wound margins over time showing wound closure dynamics in the 850‐nm PBM groups. (D,E) Quantification of (D) wound area (cm^2^) and (E) wound closure (%) over time in the 850‐nm PBM groups compared with controls (*n* = 5 independent biological experiments). (F) H&E staining of day‐12 wound sections from control and 850‐nm PBM (50 J/cm^2^) groups, with quantification of dermal gap length (*n* = 5 independent biological experiments). Upper panels show overall wound architecture; lower panels show higher‐magnification views highlighting neovascularization (red arrows) and newly formed hair follicles (yellow arrows). Scale bars, 500 µm (upper) and 100 µm (lower). (G) Masson's trichrome staining of day‐12 wound sections from control and 850‐nm PBM (50 J/cm^2^) groups, with quantification of collagen deposition (scale bar, 50 µm; *n* = 5 independent biological experiments). (H) Immunofluorescence staining for α‐SMA on day 12 with quantification of α‐SMA–positive area (scale bar, 50 µm; *n* = 5 independent biological experiments). (I) Immunohistochemical staining for CD31 and CD34 on day 12 with quantification of vessel area (scale bar, 50 µm; *n* = 5 independent biological experiments). (J) Dual immunofluorescence staining for CCR7 (M1) and CD206 (M2) on day 12, with quantification of CCR7‐ and CD206‐positive areas (scale bar, 50 µm; *n* = 5 independent biological experiments). (K,L) Representative wound photographs (K) and traced wound margins (L) on days 0, 4, 8, and 12 from mice treated with 625‐nm PBM at the indicated doses. (M,N) Quantification of (M) wound area (cm^2^) and (N) wound closure (%) over time in the 625‐nm PBM groups compared with controls (*n* = 5 independent biological experiments). (O) H&E staining of day‐12 wound sections from control and 625‐nm PBM (50 J/cm^2^) groups, with quantification of dermal gap length. Scale bars, 500 µm (overview) and 100 µm (higher magnification). (P) Masson's trichrome staining of day‐12 wound sections from control and 625‐nm PBM (50 J/cm^2^) groups, with quantification of collagen deposition (scale bar, 50 µm; *n* = 5 independent biological experiments). (Q) α‐SMA immunofluorescence on day 12 with quantification of α‐SMA–positive area (scale bar, 50 µm; *n* = 5 independent biological experiments). (R) CD31/CD34 immunohistochemical staining on day 12 with quantification of vessel area (scale bar, 50 µm; *n* = 5 independent biological experiments). (S) CCR7/CD206 immunofluorescence on day 12 with quantification of marker‐positive areas (scale bar, 50 µm; *n* = 5 independent biological experiments). Data are presented as mean ± s.d. Exact *p* values or significance levels are indicated in the figure. ^*^ *p* < 0.05 and ^***^ *p* < 0.01.

We next asked whether wavelength‐dependent macrophage polarization was associated with distinct wound‐healing outcomes in vivo. Full‐thickness excisional wounds were generated in BALB/c mice and treated with 850‐nm NIR or 625‐nm red light at 10, 30, or 50 J/cm^2^. Wound closure was monitored on days 0, 4, 8, and 12 (Figure 2A). Notably, wounds treated with 850‐nm NIR light healed much faster than those in untreated controls (Figure 2B,C). By day 4, closure in the 850‐nm NIR light‐treated group reached 46.75 ± 4.70%, 56.67 ± 4.16%, and 70.72 ± 3.17% for 10, 30, and 50 J/cm^2^, respectively, compared with that of 27.48 ± 4.13% in the controls. By day 8, closure improved to 56.87 ± 5.36%, 66.97 ±4.51%, and 89.34 ± 3.39%. By day 12, 850‐nm NIR at 50 J/cm^2^ achieved 95.28 ± 3.73% closure, compared to 68.99 ± 4.82% closure in the controls (Figure 2D,E). Histological analysis at day 12 using hematoxylin–eosin (H&E) staining revealed reduced dermal gaps (0.81 ± 0.22 mm) in the 850‐nm NIR light (50 J/cm^2^) group versus that in the controls (2.42 ± 0.46 mm) (Figure 2F). Masson's trichrome staining confirmed greater collagen deposition with 850‐nm NIR treatment (Figure 2G). Immunofluorescence for α‐SMA (myofibroblast marker) and immunohistochemistry for CD31/CD34 (endothelial cell markers) showed enhanced granulation and neovascularization in the 850‐nm NIR light (50 J/cm^2^) group, with higher α‐SMA expression (Figure 2H). This indicated the presence of more activated myofibroblasts and a superior healing response. Similarly, CD31 and CD34 staining results revealed a higher density of blood vessels in the 850‐nm NIR light (50 J/cm^2^) group, suggesting superior vascularization and matrix remodeling activity (Figure 2I). Notably, macrophages in 850‐nm NIR‐treated wounds shifted from pro‐inflammatory M1 (decreased CCR7) to anti‐inflammatory M2 phenotype (increased CD206), consistent with late‐phase wound healing (Figure 2J).

In contrast, 625‐nm red light PBM impeded healing (Figure 2K,L). By day 12, wounds treated with 625‐nm red light at 30 or 50 J/cm^2^ achieved only 54.68 ± 4.48% and 52.98 ± 4.50% closure, respectively, which was less than that achieved in the controls (71.06 ± 2.82%) (Figure 2M,N). Histologically, 625‐nm red light‐treated wounds (50 J/cm^2^) had larger dermal gaps (4.83 ± 0.47 mm) than those in the controls (2.31 ± 0.43 mm) with reduced collagen deposition, as indicated by Masson's trichrome staining (Figure 2O,P). Correspondingly, myofibroblast activation and neovascularization were suppressed (Figure 2Q,R). Macrophages in 625‐nm red light‐treated wounds retained a pro‐inflammatory M1 phenotype, with elevated CCR7 and reduced CD206 expression (Figure 2S). These findings demonstrate that 625‐nm red light irradiation sustains inflammation and delays healing, whereas 850‐nm NIR irradiation promotes timely inflammation resolution and tissue repair.

### 2.3 Mitochondrial Dynamics as Regulators of Wavelength‐Specific Macrophage Polarization

**FIGURE 3:**
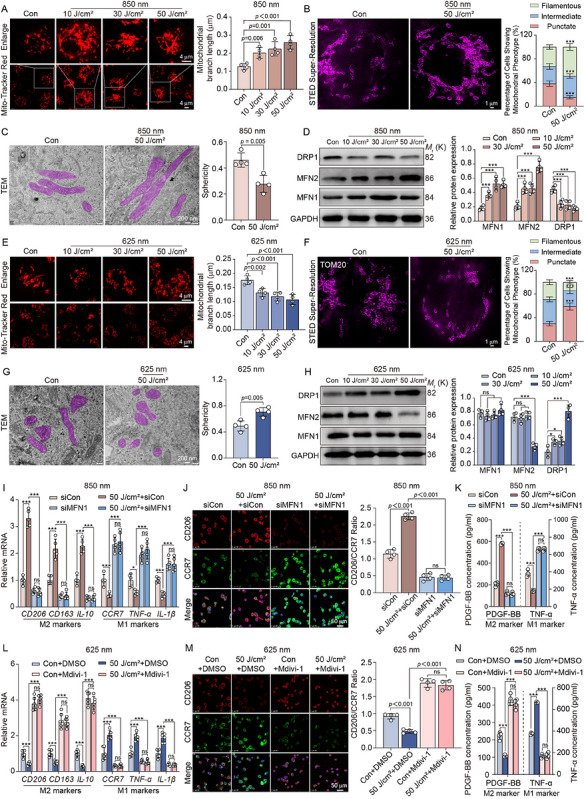
Mitochondrial dynamics mediate wavelength‐specific macrophage polarization. (A) MitoTracker Red staining of macrophages exposed to 850‐nm NIR light at 10, 30, or 50 J/cm^2^, showing a dose‐dependent increase in mitochondrial length (scale bar, 50 µm; *n* = 4 independent biological experiments). (B) STED super‐resolution images of TOM20‐labeled mitochondria after 850‐nm irradiation (50 J/cm^2^), showing a shift toward filamentous mitochondrial networks; corresponding quantification of mitochondrial morphology is shown (scale bar, 1 µm; *n* = 4 independent biological experiments). (C) TEM images of macrophages exposed to 850‐nm irradiation (50 J/cm^2^), showing elongated, rod‐like mitochondria; quantification indicates reduced mitochondrial sphericity compared with controls (scale bar, 200 nm; *n* = 4 independent biological experiments). (D) Western blot analysis of mitochondrial dynamics regulators (MFN1, MFN2, DRP1) following 850‐nm irradiation (*n* = 3 independent biological experiments). (E) MitoTracker Red staining of macrophages exposed to 625‐nm red light at 10, 30, or 50 J/cm^2^, showing reduced mitochondrial length (scale bar, 50 µm; *n* = 4 independent biological experiments). (F) STED images of TOM20‐labeled mitochondria after 625‐nm irradiation (50 J/cm^2^), showing predominantly punctate mitochondria; morphology quantification is shown (scale bar, 1 µm; *n* = 4 independent biological experiments). (G) TEM images of macrophages exposed to 625‐nm irradiation (50 J/cm^2^), showing small, round mitochondria (scale bar, 200 nm; *n* = 4 independent biological experiments). (H) Western blot analysis of MFN1, MFN2, and DRP1 following 625‐nm irradiation (*n* = 3 independent biological experiments). (I) qRT–PCR analysis of polarization‐associated genes after MFN1 knockdown in macrophages irradiated with 850‐nm NIR light (50 J/cm^2^; *n* = 5 independent biological experiments). (J) Immunofluorescence staining for CD206 (red) and CCR7 (green) under the conditions in (I), with quantification of the CD206/CCR7 fluorescence intensity ratio (scale bar, 50 µm; *n* = 4 independent biological experiments). (K) ELISA quantification of TNF‐α and PDGF‐BB in culture supernatants 24 h after MFN1 knockdown and 850‐nm irradiation (*n* = 4 independent biological experiments). (L) qRT–PCR analysis of polarization‐associated genes in macrophages exposed to 625‐nm red light (50 J/cm^2^) with or without the DRP1 inhibitor Mdivi‐1 (25 µM, 1 h; *n* = 6 independent experiment). (M) Immunofluorescence staining for CD206 and CCR7 under the conditions in (L), with quantification of the CD206/CCR7 fluorescence intensity ratio (scale bar, 50 µm; *n* = 4 independent experiment). (N) ELISA quantification of TNF‐α and PDGF‐BB in culture supernatants 24 h after Mdivi‐1 treatment and 625‐nm irradiation (*n* = 4 independent experiment). Data are presented as mean ± s.d. Exact *p* values or significance levels are indicated in the figure. ^*^ *p* < 0.05 and ^***^ *p* < 0.01; ns not significant.

Recent immunometabolism studies show that manipulating mitochondrial network dynamics directly affects macrophage polarization [32]. However, the role of PBM in macrophage polarization through mitochondria dynamics remains unclear. To address this, mitochondrial dynamics were assessed after exposure to 625‐nm red light and 850‐nm NIR light irradiations. Notably, results revealed a significant, dose‐dependent increase in mitochondrial length with 850‐nm NIR light (10–50 J/cm^2^) (Figure 3A). Super‐resolution stimulated emission depletion (STED) microscopy confirmed this shift, indicating that at 50 J/cm^2^, 850‐nm NIR light produced a more filamentous mitochondrial network rather than punctate structures (Figure 3B). Furthermore, transmission electron microscopy (TEM) showed a transition from short, rounded mitochondria to elongated, rod‐like forms after 850‐nm NIR irradiation (Figure 3C). The key regulators of mitochondrial dynamics were also assessed. Consistently, irradiation with 850‐nm NIR light upregulated fusion mediators Mitofusin 1 (MFN1) and Mitofusin 2 (MFN2) while downregulating the fission protein DRP1 (Figure 3D). In contrast, 625‐nm red light decreased mitochondrial length (Figure 3E), with STED microscopy showing predominantly fragmented (punctate) mitochondria (Figure 3F) and TEM revealing small, round mitochondria (Figure 3G). Moreover, 625‐nm red light irradiation suppressed MFN2, increased DRP1, and minimally affected MFN1 expression (Figure 3H). These results highlight the wavelength‐specific regulation of mitochondrial dynamics, with 850‐nm NIR light promoting mitochondrial fusion, whereas 625‐nm red light induces fission.

To determine whether mitochondrial fusion mediates 850‐nm NIR light‐induced M2 polarization, small interfering RNA (siRNA) was used to knock down MFN1 or MFN2 (Figure S7A,B). Under 850‐nm NIR light, MFN1/2 knockdown reduced M2‐associated gene expression and increased M1‐associated genes (Figure 3I and Figure S8A). This shift was accompanied by a reduced CD206/CCR7 ratio in immunofluorescence staining (Figure 3J and Figure S8B). Consistently, ELISA showed decreased PDGF‐BB and increased TNF‐α levels after MFN1/2 knockdown (Figure 3K and Figure S8C). These results indicate that inhibiting mitochondrial fusion shifts macrophages toward an M1 phenotype. To examine the role of mitochondrial fission in 625‐nm red light‐induced M1 polarization, cells were treated with the DRP1 inhibitor Mdivi‐1. This preserved mitochondrial length by reducing fission and drove macrophages toward an M2 phenotype, upregulating M2‐associated genes and downregulating M1 genes (Figure 3L and Figure S9). This phenotypic conversion was further reflected by a higher CD206/CCR7 ratio in immunofluorescence staining (Figure 3M). Consistently, ELISA showed increased PDGF‐BB and reduced TNF‐α levels after DRP1 inhibition (Figure 3N). Altogether, these results show that 850‐nm NIR light promotes mitochondrial fusion and drives M2 polarization, whereas 625‐nm red light enhances mitochondrial fission and drives M1 polarization. Overall, mitochondrial dynamics serve as a key mediator linking light wavelength to macrophage polarization.

### 2.4 PBM Programs Macrophage Metabolism in a Wavelength‐Dependent Manner

**FIGURE 4:**
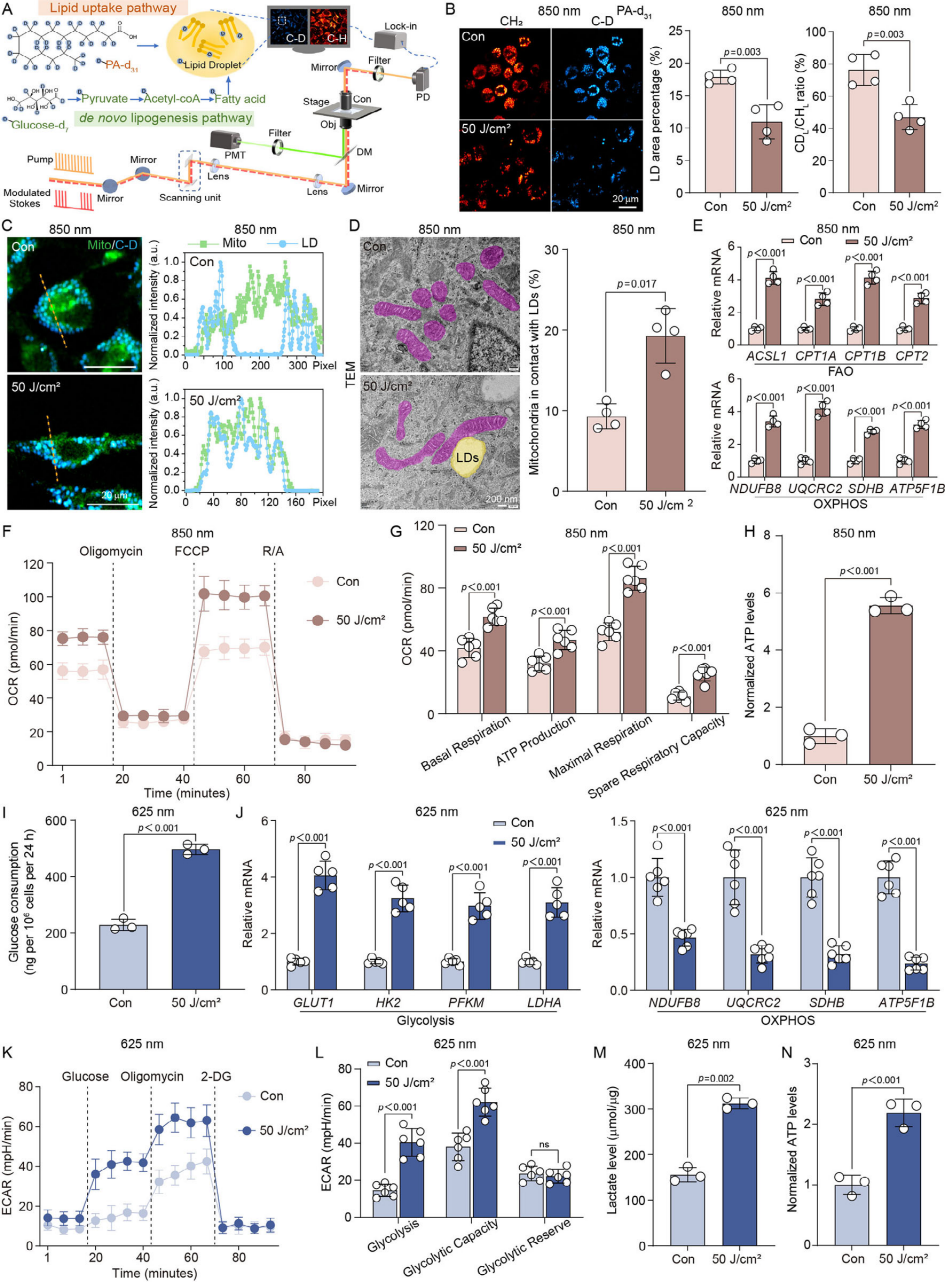
PBM programs macrophage metabolism in a wavelength‐dependent manner. (A) Experimental workflow and SRS imaging setup. Macrophages were incubated with deuterium‐labeled palmitic acid (PA‐d_31_, 25 µM) or deuterium‐labeled glucose (glucose‐d_7_, 50 µM) for 24 h before SRS imaging to quantify exogenous fatty acid incorporation and *de novo* lipogenesis in LDs. M, mirror; EOM, electro‐optic modulator; DM, dichroic mirror; SU, scanning unit; Obj, objective lens; Con, condenser; SP, short‐pass filter; PD, photodiode. (B) Representative SRS images acquired at the CH_2_ band (∼2850 cm^−1^) and C–D band (∼2107 cm^−1^) in PA‐d_31_–labeled macrophages after 850‐nm irradiation (50 J/cm^2^). Quantification of LD abundance and the C–D/CH_2_ (CD_L_/CH_L_) ratio is shown (scale bar, 20 µm; *n* = 4 independent biological experiments). (C) Merged TPEF/SRS (C–D channel) images showing increased spatial overlap between LDs and mitochondria in macrophages after 850‐nm irradiation (50 J/cm^2^) (scale bar, 20 µm). (D) TEM images showing mitochondria–LD contacts after 850‐nm irradiation (50 J/cm^2^); mitochondria and LDs are pseudocolored (magenta and yellow, respectively). Quantification of mitochondria–LD contacts is shown (scale bar, 200 nm; *n* = 4 independent biological experiments). (E) qRT–PCR analysis of FAO‐related genes (*ACSL1*, *CPT1A*, *CPT1B*, *CPT2*) and OXPHOS‐related genes (*NDUFB8*, *UQCRC2*, *SDHB*, *ATP5F1B*) after 850‐nm irradiation (50 J/cm^2^, *n* = 4 independent biological experiments). (F,G) Seahorse XF analysis of OCR after 850‐nm irradiation (50 J/cm^2^), including basal respiration, ATP‐linked respiration, maximal respiration, and spare respiratory capacity (*n* = 6 independent biological experiments). (H) Intracellular ATP levels after 850‐nm irradiation, normalized to total protein content (*n* = 3 independent biological experiments). (I) Glucose consumption after 625‐nm irradiation, normalized to total protein content (*n* = 3 independent biological experiments). (J) qRT–PCR analysis of glycolysis‐related genes (*GLUT1*, *HK2*, *PFKM*, *LDHA*) and OXPHOS‐related genes after 625‐nm irradiation (*n* = 5 independent biological experiments). (K,L) Seahorse XF analysis of ECAR after 625‐nm irradiation, including glycolysis, glycolytic capacity, and glycolytic reserve (*n* = 6 independent biological experiments). (M) Lactate production after 625‐nm irradiation (*n* = 3 independent biological experiments). (N) Intracellular ATP levels after 625‐nm irradiation, normalized to total protein content (*n* = 3 independent biological experiments). Data are presented as mean ± s.d. Exact *p* values or significance levels are indicated in the figure. ns not significant.

Mitochondrial dynamics are tightly coupled to energy metabolism [24]. Hence, the metabolic reprogramming was further examined under 625‐nm red light and 850‐nm NIR light, with a focus on three canonical inputs to macrophage polarization, namely glucose, long‐chain fatty acids, and glutamine. Glucose supports glycolysis and the pentose phosphate pathway; fatty acids are involved in fatty acid oxidation (FAO) and OXPHOS; and glutamine replenishes the tricarboxylic acid cycle via α‐ketoglutarate [33]. Accordingly, wavelength‐dependent uptake and utilization of these substrates were quantified during polarization. In mammalian cells, glucose can undergo *de novo* lipogenesis to form fatty acids, which, when combined with exogenous fatty acids, can be esterified and stored in lipid droplets (LDs), which serve as dynamic organelles for lipid storage and energy release [34]. To assess LD use as an energy source during 850‐nm NIR light‐induced polarization, stimulated Raman scattering (SRS) microscopy was employed. SRS microscopy is a highly sensitive, chemical imaging technique for quantitative mapping in single cells in situ at submicron resolution and minimal photodamage [35, 36]. SRS detects vibrational signatures of metabolic tags such as deuterium, providing a label‐free approach to monitoring lipid synthesis and uptake [37]. Notably, SRS microscopy revealed a distinct vibrational frequency for the CH_2_ band at 2850 cm^−1^, identifying LDs within individual cells. SRS signals from the C‐D band at 2107 and 2135 cm^−1^ denoted newly synthesized lipids in LDs after cells were fed with deuterium‐labeled palmitic acid (PA‐d_31_) and deuterium‐labeled glucose (glucose‐d_7_), respectively. The ratio of C–D signal to C–H signal reflected newly synthesized LD content, further indicating LD turnover (Figure 4A). Macrophages fed with PA‐d_31_ showed reduced total LD content after 850‐nm NIR light irradiation, indicating lipid consumption during M2 polarization (Figure 4B). In contrast, macrophages fed with glucose‐d_7_ to probe *de novo* lipogenesis revealed that 850‐nm NIR light irradiation did not significantly change LD content (Figure S10). These results suggest that 850‐nm NIR light‐induced M2 polarization relies primarily on exogenous fatty acid uptake and oxidation (via LD consumption) rather than on *de novo* synthesis.

Upon breakdown, LDs release free fatty acids for mitochondrial FAO. Hence, LD–mitochondria spatial relationships were examined under 850‐nm NIR light exposure through SRS and two‐photon excited fluorescence (TPEF) imaging. These analyses revealed numerous LDs nearly overlapping with mitochondria in 850‐nm NIR‐irradiated macrophages (Figure 4C). TEM confirmed increased mitochondria–LD interactions following 850‐nm NIR exposure (Figure 4D). We hypothesized that this increase in LD–mitochondria interaction enhances FAO in mitochondria. Interestingly, the expression of FAO‐ and OXPHOS‐related genes (including *ACSL1*, *CPT1A*, *CPT1B*, *CPT2*, *NDUFB8*, *UQCRC2*, *SDHB*, and *ATP5F1B*) significantly increased following 850‐nm NIR irradiation (Figure 4E). Consistently, CPT1A protein expression was also increased after 850‐nm irradiation (Figure S11). Furthermore, Seahorse metabolic flux assays revealed markedly higher oxygen consumption rates (OCRs), including basal respiration, maximal respiration, ATP production, and spare respiratory capacity, in 850‐nm NIR‐irradiated macrophages (Figure 4F,G). These results indicate that mitochondrial OXPHOS is strongly upregulated by PBM under 850‐nm NIR light. Consistently, intracellular ATP levels were markedly increased following irradiation with 850‐nm NIR light (Figure 4H). Assessment of the effects of 850‐nm NIR light on other metabolic pathways revealed no significant changes in glucose uptake, glycolysis‐related gene expression (*GLUT1*, *HK2*, *PFKM*, *LDHA*), glutamine consumption, and glutaminase (GLS) activity (Figures S12A,B and S13A,B). These results show that 850‐nm NIR light‐induced M2 polarization does not rely on increased glucose or glutamine metabolism and predominantly depends on fatty acid metabolism.

Analysis of metabolic changes during 625‐nm red light‐induced M1 polarization revealed a marked increase in glucose consumption (Figure 4I), along with upregulated glycolytic genes and downregulated OXPHOS genes (Figure 4J). Consistently, HK2 protein expression was also increased after 625‐nm irradiation (Figure S14). Basal extracellular acidification rate and lactate production increased significantly following 625‐nm red light irradiation (Figure 4K–M), indicating preferential pyruvate‐to‐lactate conversion (a hallmark of glycolytic metabolism). Furthermore, intracellular ATP levels were substantially increased (Figure 4N), indicating increased energy generation via glycolysis and subsequent rapid response of M1 polarization. The effects of fatty acids or glutamine on metabolism under irradiation with 625‐nm red light were examined, and LD analysis (PA‐d_31_ or glucose‐d_7_) showed no notable changes (Figure S15A,B), indicating fatty acid uptake or lipogenesis as non‐primary energy sources. Similarly, glutamine consumption and GLS activity remained unchanged under 625‐nm red light exposure (Figure S16A,B). Collectively, these data confirm that PBM with 625‐nm red light drives a primarily glycolytic program rather than involving fatty acid or glutamine pathways.

In summary, specific wavelengths distinctly reprogram macrophage metabolism to drive polarization, with 850‐nm NIR light promoting OXPHOS‐dominant, FAO‐fueled M2 metabolism and 625‐nm red light driving glycolysis‐dominant M1 metabolism.

### 2.5 Metabolic Inhibition Reshapes Mitochondrial Dynamics to Drive Compensatory Metabolic Shifts and Macrophage Polarization

**FIGURE 5:**
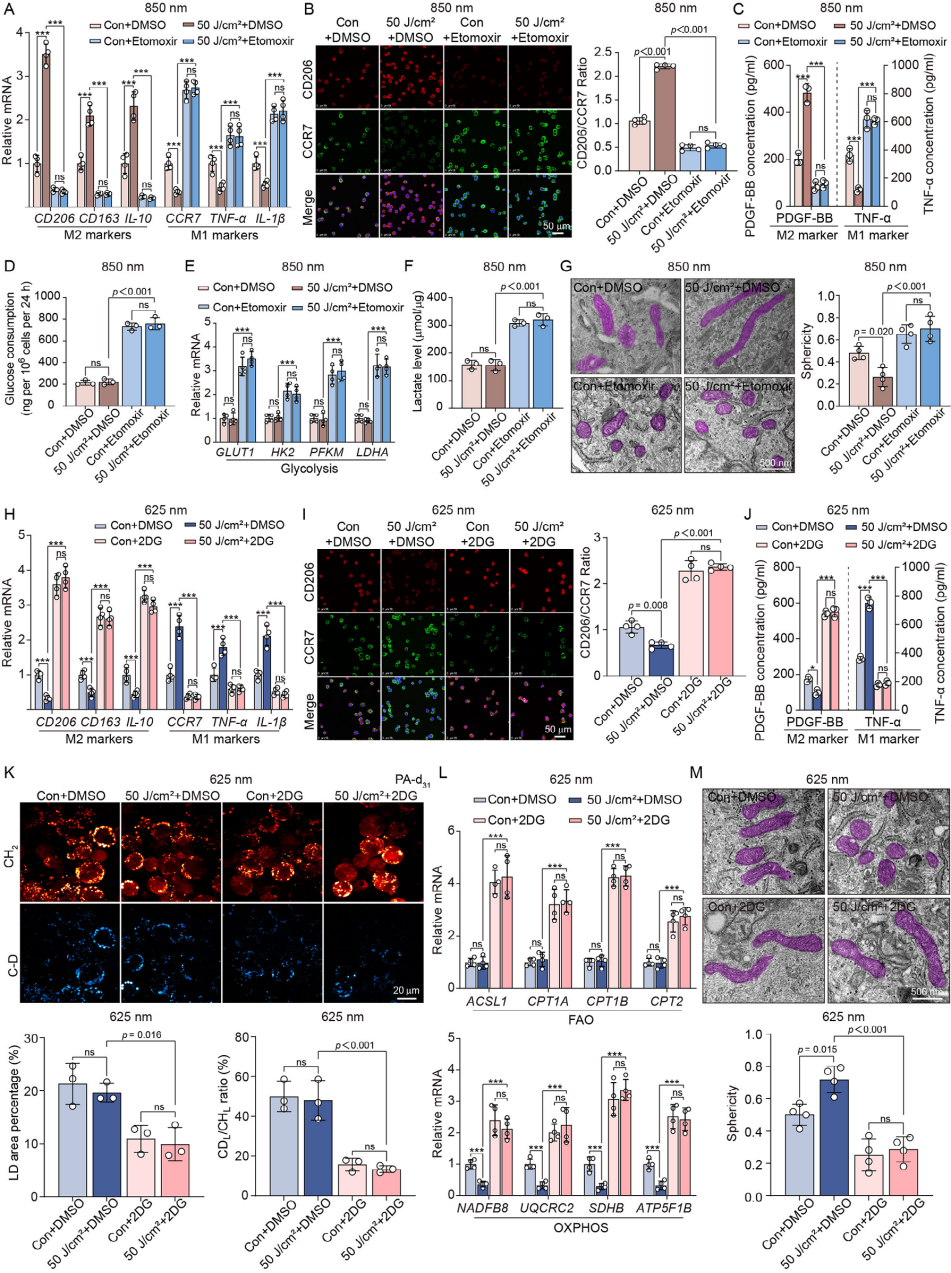
Metabolic inhibition reshapes mitochondrial dynamics and drives compensatory metabolic switching to reprogram macrophage polarization. (A–G) Macrophages were pretreated with the FAO inhibitor etomoxir (200 µM, 3 h) and then irradiated with 850‐nm NIR light (50 J/cm^2^). (A) qRT–PCR analysis of polarization‐associated genes (*n* = 4 independent biological experiments). (B) Immunofluorescence staining for CD206 (red) and CCR7 (green), with quantification of the CD206/CCR7 fluorescence intensity ratio (scale bar, 50 µm; *n* = 4 independent biological experiments). (C) ELISA quantification of TNF‐α and PDGF‐BB in culture supernatants 24 h after treatment (*n* = 3 independent biological experiments). (D) Glucose consumption normalized to total protein content (*n* = 3 independent biological experiments). (E) qRT–PCR analysis of glycolysis‐related genes (*n* = 4 independent biological experiments). (F) Lactate production (*n* = 3 independent biological experiments). (G) TEM images of mitochondrial ultrastructure with quantification of mitochondrial sphericity (scale bar, 500 nm; *n* = 4 independent biological experiments). (H–M) Macrophages were pretreated with the glycolysis inhibitor 2DG (10 mM, 1 h) and then irradiated with 625‐nm red light (50 J/cm^2^). (H) qRT–PCR analysis of polarization‐associated genes (*n* = 4 independent biological experiments). (I) Immunofluorescence staining for CD206 (red) and CCR7 (green), with quantification of the CD206/CCR7 fluorescence intensity ratio (scale bar, 50 µm; *n* = 4 independent biological experiments). (J) ELISA quantification of TNF‐α and PDGF‐BB in culture supernatants 24 h after treatment (*n* = 3 independent biological experiments). (K) SRS imaging acquired at the CH_2_ band (∼2850 cm^−1^) and C–D band (∼2107 cm^−1^), with quantification of LD abundance and the C–D/CH_2_ (CD_L_/CH_L_) ratio (scale bar, 20 µm; *n* = 3 independent biological experiments). (L) qRT–PCR analysis of FAO‐ and OXPHOS‐related genes (*n* = 4 independent biological experiments). (M) TEM images of mitochondrial ultrastructure with quantification of mitochondrial sphericity (scale bar, 500 nm; *n* = 4 biological independent biological experiments). Data are presented as mean ± s.d. Exact *p* values or significance levels are indicated in the figure. ^*^ *p* < 0.05 and ^***^ *p* < 0.01; ns not significant.

To further examine the association between metabolism and polarization, the role of FAO in 850‐nm NIR light‐induced M2 polarization was investigated. Macrophages were treated with etomoxir, a carnitine palmitoyltransferase‐1 (CPT1) inhibitor essential for transporting long‐chain fatty acids into mitochondria for FAO. Under 850‐nm irradiation, etomoxir suppressed M2‐related genes and increased M1‐related genes (Figure 5A). At the protein level, etomoxir decreased the CD206/CCR7 ratio (Figure 5B) and reduced PDGF‐BB while increasing TNF‐α secretion (Figure 5C), confirming that active FAO sustains 850‐nm NIR‐induced M2 polarization. As blocking FAO might force cells to utilize glycolysis, glycolytic activity post‐etomoxir treatment was assessed. Etomoxir‐treated macrophages showed increased glucose consumption, upregulated glycolytic genes (*GLUT1*, *HK2*, *PFKM*, and *LDHA*), and elevated lactate production (Figure 5D–F). These results suggest that inhibiting FAO under 850‐nm NIR light triggers compensatory glycolysis activation, supporting an M1 phenotype. Consistent with this shift, mitochondrial imaging revealed increased sphericity (more rounded mitochondria) post‐etomoxir treatment under 850‐nm NIR light, indicating a fusion‐to‐fission shift (Figure 5G).

Additionally, the role of glycolysis in 625‐nm red light‐induced M1 polarization was examined using 2‐deoxy‐D‐glucose (2DG), a hexokinase inhibitor. Under 625‐nm irradiation, 2DG suppressed M1‐related genes and increased M2‐related genes (Figure 5H). At the protein level, 2DG increased the CD206/CCR7 ratio (Figure 5I) and elevated PDGF‐BB while reducing TNF‐α secretion (Figure 5J), confirming that active glycolysis sustains 625‐nm red light‐induced M1 polarization. We then tested if blocking glycolysis promoted FAO. SRS microscopy revealed significantly reduced LD content with 2DG under 625‐nm red light (Figure 5K), indicating increased LD consumption following glycolysis inhibition. Correspondingly, FAO‐ and OXPHOS‐related genes were upregulated in 2DG‐treated macrophages under 625‐nm red light (Figure 5L). These results show that inhibiting glycolysis under 625‐nm red light shifts metabolism toward FAO, favoring M2 polarization. Mitochondrial imaging confirmed this shift, with 2DG‐treated macrophages showing reduced mitochondrial sphericity (more elongated mitochondria), indicating a fission‐to‐fusion shift (Figure 5M). Collectively, these findings demonstrate that PBM combined with metabolic interventions redirects macrophage polarization. Inhibiting FAO during 850‐nm NIR light drives a glycolytic, M1 macrophage phenotype, whereas inhibiting glycolysis during 625‐nm red light promotes FAO and an M2 macrophage phenotype. This reveals a complex, exploitable interplay between wavelength‐specific metabolic programming and mitochondrial dynamics. Overall, strategic metabolic manipulation under defined PBM wavelengths induces compensatory shifts, offering new avenues for tailored immunometabolic therapies in wound healing.

## 3 Discussion

This study reveals that light at defined wavelengths can act as a targeted switch for macrophage immunometabolism, thereby determining macrophage polarization and function. Herein, a custom‐built irradiation system was used to show that distinct wavelengths elicit discrete macrophage phenotypes independently of thermal effects. Specifically, 850‐nm NIR irradiation induced an anti‐inflammatory M2 macrophage phenotype, characterized by enhanced fatty acid uptake, tighter LD–mitochondria coupling, increased mitochondrial fusion, and elevated FAO, which were associated with accelerated tissue repair in a mouse wound‐healing model. In contrast, 625‐nm red light shifted macrophages toward a pro‐inflammatory M1 phenotype, marked by higher glucose uptake, increased glycolytic flux and lactate secretion, pronounced mitochondrial fission, and delayed wound healing. Crucially, pharmacological inhibition of the dominant metabolic pathway at each wavelength, FAO inhibition under 850‐nm NIR and glycolysis inhibition under 625‐nm red light, reversed polarization phenotypes. Altogether, these findings position metabolism as a key mediator of PBM‐driven macrophage polarization, suggesting that tuning light wavelength offers an effective strategy to modulate immune responses and accelerate tissue repair.

PBM is a well‐known, promising, non‐invasive strategy for modulating immune function and macrophage behavior [38]. However, reported immunomodulatory outcomes vary widely across studies, largely owing to inconsistent irradiation parameters [39]. Prior research emphasizes that careful wavelength and dose selection is critical, with most studies using red or NIR light in the 600–1000 nm “optical therapeutic window” to maximize tissue penetration and cellular signaling [40]. The red (600–700 nm) and NIR (780–950 nm) irradiation within this range offer deeper tissue penetration and potent effects on cellular photoreceptors [14, 41]. However, heterogeneity in light sources and dosimetry parameters, including wavelength, energy density (fluence), exposure duration, beam profile, spot size, and temperature control, likely explains much of the discrepancy in immune responses [39, 42]. Beyond variation in irradiation parameters, an additional limitation of conventional PBM setups is that the actual optical input delivered to cells or tissues is often not rigorously controlled. Methodological studies have emphasized that incomplete reporting and insufficient control of key PBM delivery parameters, including irradiance, beam area, spot size, and exposure conditions, limit reproducibility and cross‐study comparison [43, 44]. In addition, PBM devices in routine use may show deviations in actual output power and beam diameter from manufacturer‐declared specifications, and non‐uniform Gaussian‐type beam profiles can generate spatially uneven dose delivery across the treated field [45]. Such variability is particularly problematic for mechanistic studies, because non‐uniform irradiation can lead to uneven cellular stimulation and obscure true wavelength‐dependent biological effects [46]. In contrast, our custom‐designed PBM platform was developed specifically to minimize these methodological sources of variation. The system enables user‐defined control of wavelength, irradiance, and exposure duration under incubator‐compatible conditions, incorporates closed‐loop feedback regulation to maintain irradiance within ±5% of the target setpoint, and uses a light‐mixing rod together with a condenser lens assembly to achieve >85% spatial uniformity across a 5 × 5 cm illumination field. This design reduces technical variability in optical delivery and improves the rigor of wavelength‐dependent analysis. Under these conditions, clear wavelength‐specific effects were observed. Notably, 625‐nm red light skewed THP‐1‐derived macrophages toward an M1 phenotype, whereas 850‐ and 935‐nm NIR light favored an M2 phenotype. These results align with prior studies reporting pro‐inflammatory responses to shorter‐wavelength red light and pro‐resolving effects from NIR irradiation [13, 47, 48]. Further validation of this wavelength‐dependent polarization pattern in primary mouse BMDMs and a mouse wound‐healing model confirmed the robustness of the above‐mentioned results across both in vitro and in vivo settings. Beyond wavelength, higher doses (energy densities > 50 J/cm^2^) were found to significantly reduce macrophage viability, consistent with the biphasic dose–response of PBM [49]. This behavior (described by the Arndt–Schulz curve) indicates that even optimal wavelengths require precise dosing to preserve cell viability and achieve immunomodulatory benefits [42, 50]. Together, these results highlight the importance of both wavelength and dose in obtaining reliable PBM outcomes.

The present findings further support a role for mitochondrial dynamics in PBM‐mediated macrophage polarization. Mitochondrial fusion and fission are increasingly recognized as being closely linked to macrophage metabolic state and inflammatory phenotype, with fusion generally associated with preserved respiratory function, oxidative metabolism, and reparative programs, whereas fission is more often associated with fragmented mitochondrial networks, glycolytic rewiring, and pro‐inflammatory activation [32, 51]. Notably, mitochondria are major cellular photoacceptors for red and NIR irradiation and absorb photons in this spectral range to modulate mitochondrial function and downstream signaling [28]. In this context, our data show that 850‐nm NIR light promoted mitochondrial fusion in macrophages, as indicated by increased mitochondrial length and upregulation of MFN1 and MFN2, resulting in a more elongated and interconnected mitochondrial network consistent with the bioenergetic demands of M2 macrophages [52]. By contrast, 625‐nm red light induced mitochondrial fission, as reflected by reduced mitochondrial length and increased DRP1 expression, a pattern characteristic of classically activated M1 macrophages [32]. Together, these findings place our results within an emerging framework in which mitochondrial fusion is linked to reparative macrophage programs, whereas mitochondrial fission is associated with pro‐inflammatory activation [53], and further suggest that wavelength‐specific PBM can function as an upstream physical cue that biases macrophage phenotype through differential regulation of mitochondrial dynamics.

Given the close coupling between mitochondrial organization and cellular metabolism [54], we next examined whether these wavelength‐dependent structural changes were accompanied by distinct metabolic programs. Macrophage polarization is tightly coupled to metabolic state, and the present findings further connect wavelength‐specific PBM to this immunometabolic framework. Under 850‐nm NIR irradiation, which promoted mitochondrial fusion, macrophages adopted an OXPHOS‐ and FAO‐dominant metabolic profile consistent with an M2‐like phenotype [55]. This shift was supported by enhanced LD–mitochondria coupling, increased OCR and ATP production, and upregulation of FAO‐ and OXPHOS‐related genes. By contrast, 625‐nm red irradiation promoted a glycolysis‐dominant metabolic program, as evidenced by increased glucose consumption, elevated lactate production, enhanced glycolytic flux, and upregulation of glycolysis‐related genes, consistent with an M1‐like phenotype [56]. Notably, these metabolic programs were closely aligned with the corresponding changes in mitochondrial organization, further supporting a tight coupling between mitochondrial dynamics and metabolic pathway choice during macrophage phenotype regulation. Together, these data support a model in which wavelength‐specific PBM biases macrophage polarization by coordinating mitochondrial dynamics with metabolic reprogramming.

To strengthen the causal inference that metabolism drives polarization (rather than merely accompanying it), targeted metabolic interventions under different conditions were performed. The results revealed that PBM‐induced polarization states are maintained by dynamic, compensatory metabolic networks. Pharmacological blocking of FAO during 850‐nm NIR irradiation with etomoxir (a CPT1a inhibitor) prevented M2 phenotype sustenance, as indicated by a decrease in M2 markers (CD206, IL10) and increased M1 markers (CCR7, TNF‐α). FAO blockade also triggered elevated glucose utilization, upregulation of glycolytic enzymes, and increased lactate secretion, effectively shifting metabolism toward glycolysis. Consistently, the fused mitochondrial network was observed to shift toward a more fragmented, punctate morphology. These changes indicate that, under 850‐nm NIR light, FAO‐driven OXPHOS loss induces macrophages to activate glycolysis as a backup ATP‐generating pathway, ultimately leading to an M2‐to‐M1 phenotype shift. In essence, the anti‐inflammatory M2 state enforced by 850‐nm NIR light substantially depends on mitochondrial oxidative metabolism, which, when pharmacologically disrupted, leads macrophages to default to a pro‐inflammatory, glycolysis‐supported state. The present observations are consistent with previous studies reporting that macrophages exhibit considerable metabolic plasticity and can recruit glycolysis when OXPHOS is impaired [57]. Conversely, inhibiting glycolysis during 625‐nm red light exposure (using 2‐DG) blunted M1 phenotype (evidenced by reduced inflammatory cytokine levels) and elevated M2 markers. This forced macrophages to activate alternative fuel sources, as observed by depletion of intracellular LDs (indicating utilization of stored fatty acids) and upregulation of FAO/OXPHOS genes. This metabolic switch was accompanied by a notable change in organelle morphology. Mitochondria shifted from fragmented to elongated/fused forms following glycolysis suppression. This shows that under glycolysis blockade, M1 macrophages activate FAO respiration to meet their energy needs, thereby reprogramming toward an M2 phenotype. Collectively, these findings show that PBM‐imposed mitochondrial and metabolic states can be dynamically rewired through substrate flexibility and metabolic crosstalk. Furthermore, the direction of this compensatory reprogramming, toward glycolysis or FAO, ultimately determines the polarization fate of the macrophages.

This study has several limitations. The mechanistic framework proposed here was established primarily in THP‐1‐derived macrophages and further supported in mouse BMDMs and a wound‐healing model; broader validation in primary human systems and additional disease contexts will be important. In addition, although representative protein‐level validation was included, the upstream events linking wavelength‐specific PBM to mitochondrial and metabolic remodeling remain to be further define. Finally, this study includes a broad set of endpoints across molecular, imaging, metabolic, and functional domains. Although post‐hoc multiple‐comparison corrections were applied within individual analyses where appropriate, broader multiplicity across experiments remains only partially mitigated and should therefore be considered when interpreting individual statistical results. Accordingly, individual *p* values should be interpreted together with the corresponding effect‐size estimates and the overall consistency of the biological patterns observed across independent assays, rather than as isolated findings.

## 4 Conclusion

**FIGURE 6:**
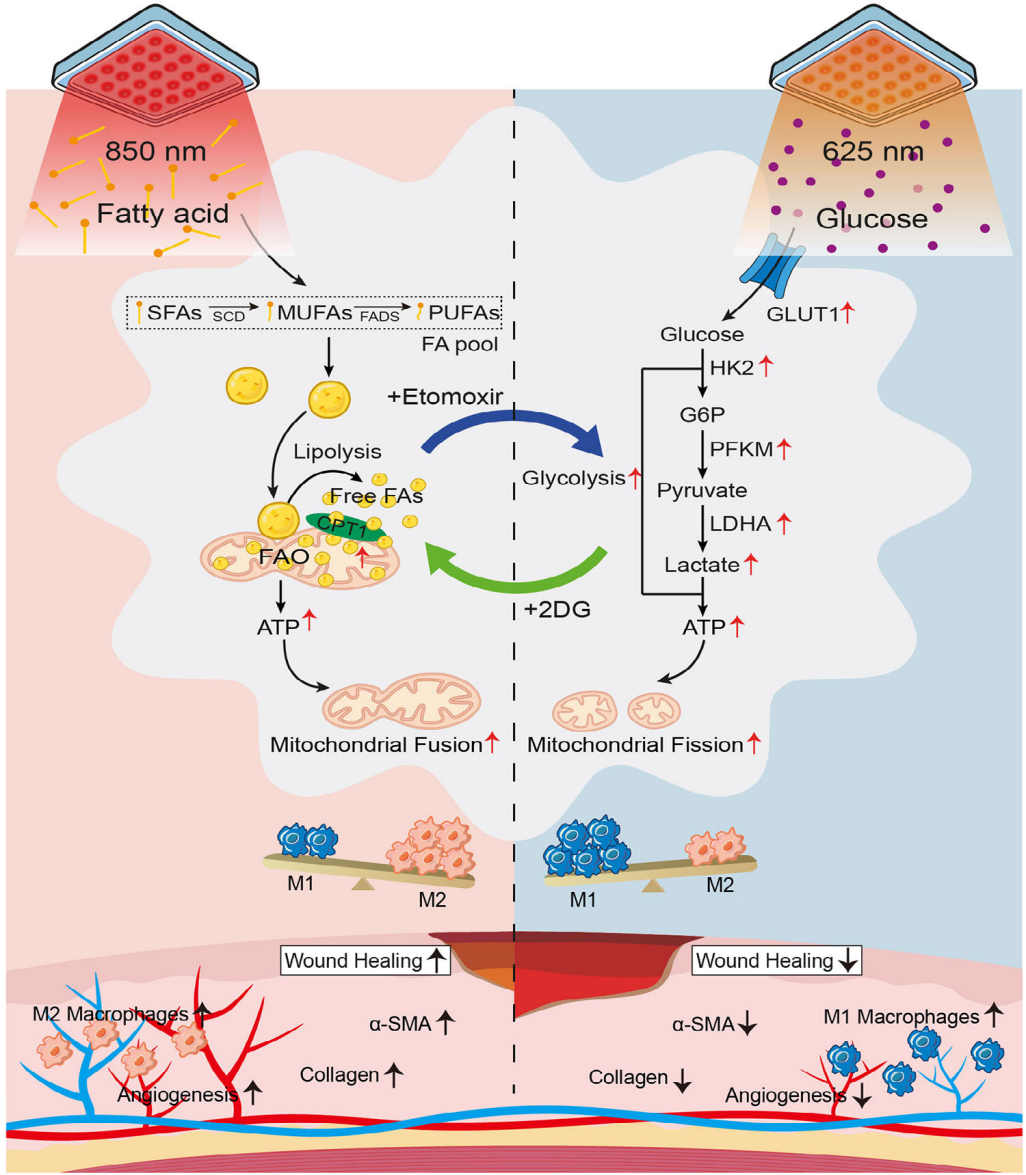
Summary of wavelength‐dependent mitochondrial and metabolic regulation of macrophage polarization by PBM. Schematic overview illustrating that 850‐nm NIR PBM promotes mitochondrial fusion, LD utilization, and FAO/OXPHOS‐dominant metabolism, thereby driving M2 polarization and enhancing wound repair, whereas 625‐nm red PBM favors mitochondrial fission and glycolysis‐dominant metabolism, thereby sustaining M1 polarization and delaying wound healing.

Here, we define photo‐immunometabolism as a wavelength‐dependent PBM framework in which light reconfigures mitochondrial dynamics and metabolic programs to influence immune cell function and fat. This framework highlights that light is not merely an exogenous stimulus, but also a targeted bioenergetic cue that can reprogram immune metabolism. Beyond this conceptual advance, our findings provide a rationale for the design of tailored PBM therapies to selectively regulate immune responses. For example, 850‐nm NIR irradiation may promote pro‐resolving M2 macrophage polarization in chronic wounds, ischemic injury, or autoimmune diseases, thereby enhancing tissue repair and anti‐inflammatory effects. In contrast, appropriately dosed 625‐nm red light may strengthen M1‐associated microbicidal and tumoricidal functions in conditions such as infection and cancer. By linking photon wavelength to immunometabolic fate, this study provides a foundation for precision light‐based therapies that fine‐tune immune responses to improve healing and disease outcomes (Figure 6).

## 5 Experimental Section

### 5.1 Cell Culture

Human THP‐1 monocyte cell line (CAS no. TIB‐202, ATCC) were cultured in RPMI‐1640 medium (CAS no. 11875093, Gibco) supplemented with 10% fetal bovine serum (FBS; Gibco) and 1% penicillin–streptomycin (P/S; CAS no. 15140122, Gibco) at 37°C in a humidified atmosphere containing 5% CO_2_. Cells were passaged every 2–3 days using fresh medium. To induce macrophage differentiation, THP‐1 cells were seeded at 1 × 10^6^ cells/mL and treated with 100 ng/mL phorbol 12‐myristate 13‐acetate (PMA; CAS no. P8139, Sigma) for 48 h. For M1 polarization, differentiated macrophages were stimulated with 20 ng/mL IFN‐γ (CAS no. AF‐300‐02, PeproTech) and 100 ng/mL LPS (CAS no. L2880, Sigma) for 48 h. For M2 polarization, macrophages were treated with 20 ng/mL IL‐4 (CAS no. 200‐04, PeproTech) and 20 ng/mL IL‐13 (CAS no. 200‐13, PeproTech) for 48 h.

BMDMs were isolated from femurs and tibias of 6–8‐week‐old male BALB/c mice. Bone marrow was flushed out with PBS, and red blood cells were lysed using RBC lysis buffer (CAS no. RT122, Tiangen). The remaining cells were cultured in Dulbecco's Modified Eagle Medium (DMEM; CAS no. 11965092, Gibco) supplemented with 10% FBS, 1% P/S, and 20 ng/mL mouse macrophage colony‐stimulating factor (M‐CSF; CAS no. 416‐ML‐010, R&D Systems) for 7 days. Medium was replaced with fresh differentiation medium on days 3 and 5. Mature macrophages were harvested on day 7.

Human fibroblasts were isolated from foreskin tissue samples collected during routine circumcision of pediatric patients, with informed consent obtained from the legal guardians. Tissues were minced into small fragments and enzymatically digested. Cells were cultured in DMEM supplemented with 10% FBS and 1% P/S at 37 °C in 5% CO_2_. Cells from passages 3–5 were used for all experiments.

### 5.2 PBM Irradiation Setup

A custom‐designed experimental apparatus was developed to deliver precisely controlled, stable, and uniform PBM over extended durations. The apparatus integrates 24 different wavelengths into a single optical platform, ranging from ultraviolet‐C (278 nm) to NIR (1577 nm). At each selected wavelength, its irradiance can be precisely tuned in the range of 5 to 100 mW/cm^2^. Except for the controller and external power driver, the entire irradiation module is housed inside a standard cell incubator. An electrical connector mounted on the incubator's side wall links the external driver to the internal LED array, thereby avoiding cable interference with the door mechanism. The control system enables long‐term PBM exposure in live‐cell experiments. Light output is regulated through a closed‐loop feedback mechanism, which compensates for thermal‐induced efficiency losses in the LEDs. This real‐time power control ensures irradiance stability and minimizes nonlinearity in the photo‐electro‐thermal characteristics of the system. The control architecture was designed using robust control synthesis to mitigate both system nonlinearities and external optical disturbances. Irradiance is continuously monitored via an integrated photodiode positioned along the central axis of the optical module and aligned directly beneath the cell culture area. The closed‐loop stabilized output profiles for the three selected wavelengths in this work, i.e., 625, 850, and 935 nm. To ensure spatial uniformity of illumination, the system employs a light‐mixing module and a condenser lens array, achieving over 85% irradiance uniformity across a 5 cm × 5 cm illumination area at a reference intensity of 50 mW/cm^2^. PBM exposure levels of 10, 30, 50, 70, and 90 J/cm^2^ were selected based on previously reported effective dose ranges (10–100 J/cm^2^). During irradiation, culture dish lids were removed, and the culture medium was temporarily replaced with PBS to prevent the generation of toxic photoproducts. Immediately following irradiation, PBS was replaced with complete culture medium for continued incubation.

### 5.3 Cell Viability Assay

Cell viability after PBM was assessed using the Cell Counting Kit‐8 (CCK‐8; CAS no. C0037, Beyotime) according to the manufacturer's instructions. THP‐1 cells were seeded at 5 × 10^3^ cells per well in 96‐well plates and differentiated into macrophages by treatment with PMA 100 ng/mL for 48 h. Differentiated macrophages were then irradiated with 625‐, 850‐, or 935‐nm light at energy density of 10, 30, 50, 70, or 90 J/cm^2^, with non‐irradiated cells serving as controls. At 24 h post‐irradiation, CCK‐8 reagent was added to each well and incubated for 4 h in the dark. Absorbance at 450 nm was measured using a microplate reader (ThermoFisher). Background absorbance (medium‐only wells) was subtracted, and optical density values were normalized to the non‐irradiated control to calculate percent viability.

### 5.4 Quantitative Real‐Time PCR

Total RNA was extracted using TRIzol reagent (Invitrogen) according to the manufacturer's instructions. RNA concentration and purity were assessed using an ND‐1000 spectrophotometer. For each sample, 2 µg of total RNA was reverse‐transcribed into cDNA. qRT–PCR was performed on a QuantStudio 3 Real‐Time PCR System (Applied Biosystems) using SYBR Green Supermix (CAS no. 639676, Takara). Relative mRNA expression levels were calculated using the 2^−ΔΔCt^ method, with *β‐actin* (*ACTB*) as the internal reference gene. Primer sequences were synthesized by Sangon and are provided in Table S5.

### 5.5 Immunofluorescence Staining

Cells were fixed in 4% paraformaldehyde (Sigma‐Aldrich, 16005) for 20 min at room temperature and then washed three times with PBS. Cells were subsequently permeabilized with 0.2% Triton X‐100 for 5 min and washed again with PBS. Non‐specific binding was blocked with 5% BSA in PBS for 2 h at room temperature. Cells were incubated overnight at 4°C with primary antibodies against CD206 (CAS no. ab64693, Abcam, 1:250) and CCR7 (CAS no. ab253187, Abcam, 1:250). After washing, cells were incubated with secondary antibodies conjugated to Alexa Fluor 488 (CAS no. ab150113, Abcam, 1:500) or Alexa Fluor 647 (CAS no. ab150115, Abcam, 1:500) for 1 h at room temperature. Nuclei were counterstained with 4′,6‐diamidino‐2‐phenylindole (DAPI; CAS no. D1306, Invitrogen, 1:2000). Images were acquired using a Leica TCS SP8 or an Andor Dragonfly 500 confocal microscope. Fluorescence intensity and co‐localization (e.g., CD206/CCR7 ratio) were quantified using Fiji (version 2.1.0/1.53c).

### 5.6 ELISA

Cytokine concentrations in cell culture supernatants were quantified using sandwich ELISA kits according to the manufacturers’ instructions: human PDGF‐BB (CAS no. DBB00, R&D Systems) and TNF‐α (CAS no. DTA00D, R&D Systems), and mouse IL‐10 (CAS no. M1000B, R&D Systems) and TNF‐α (CAS no. MTA00B, R&D Systems). Culture supernatants were collected, clarified by centrifugation, and applied to antibody‐coated 96‐well plates along with serially diluted standards. After incubation and washing, HRP‐conjugated detection reagents and substrate solution were added sequentially. Absorbance at 450 nm was measured using a microplate reader. Standard curves were generated in Prism 9 (version 9.5.1), and cytokine concentrations were calculated by interpolation after subtraction of background absorbance from medium‐only wells.

### 5.7 Temperature‐Matched Control Experiments

To distinguish wavelength‐specific photobiological effects from potential thermal effects, medium temperature was monitored in real time during PBM exposure using a thermocouple probe (CAS no. IT‐23, Physitemp Instruments). Based on the measured temperature elevation profiles, temperature‐matched control groups were established at 37.5, 38.0, and 38.5°C under light‐free conditions. Samples were maintained at the corresponding temperatures using a thermostatic heating plate placed inside the CO_2_ incubator (CAS no. 3951, Thermo Fisher Scientific), which was set to the same target temperature. Each temperature‐matched condition was maintained for the same duration as the corresponding PBM exposure, after which downstream analyses were performed at the same post‐treatment time points as in the PBM groups.

### 5.8 Transwell Migration Assay

Fibroblast migration was assessed using a Transwell assay. Fibroblasts were seeded into the upper chambers of Transwell inserts at 1 × 10^6^ cells/mL. PBM‐pretreated macrophages (625, 850, or 935 nm; 10, 30, or 50 J/cm^2^) were plated in the lower chambers and served as chemoattractant cells. After 24 h of co‐culture, non‐migrated cells on the upper surface of the membrane were gently removed with a cotton swab. Cells that migrated to the lower surface were fixed and stained with 0.5% crystal violet (CAS no. C0121, Beyotime) for 1 h, washed with PBS, and imaged. Migrated cells were quantified using Fiji (version 2.1.0/1.53c).

### 5.9 EdU Incorporation Assay

Fibroblast proliferation in response to macrophage‐conditioned media was assessed using the iClick EdU Andy Fluor 488 Imaging Kit (CAS no. A003, ABP Biosciences) according to the manufacturer's instructions. THP‐1 monocytes (1 × 10^6^ cells per well in 6‐well plates) were differentiated into macrophages by treatment with PMA (100 ng/mL) for 48 h. Differentiated macrophages were either left untreated or irradiated with PBM at 625, 850, or 935 nm at energy density of 10, 30, or 50 J/cm^2^. Following irradiation, macrophages were cultured for an additional 24 h, and conditioned media were collected from each group and applied to fibroblasts for 48 h. Fibroblasts were then incubated with EdU (10 µM) for 4 h, fixed in 4% paraformaldehyde for 10 min, and washed with PBS containing 3% BSA. Cells were permeabilized with 0.5% Triton X‐100 for 20 min and processed with the iClick reaction cocktail for 30 min, followed by DAPI nuclear counterstaining. Fluorescence images were acquired using a Leica TCS‐SP8 confocal microscope. The EdU incorporation rate was calculated as (EdU‐positive nuclei / total nuclei)  ×  100%.

### 5.10 Mito‐Tracker Red Staining

Mitochondrial morphology was visualized using MitoTracker Red (CAS no. C1035, Beyotime). Macrophages were incubated with MitoTracker Red at the manufacturer‐recommended working concentration for 10 min at 37°C, washed with PBS, and maintained in fresh DMEM for imaging. Live‐cell confocal imaging was performed using an Andor Dragonfly 500 system. Quantitative analysis of mitochondrial network features (e.g., total branch length per mitochondrion) was performed in Fiji (version 2.1.0/1.53c) using the MiNA (Mitochondrial Network Analysis) plugin.

### 5.11 STED Super‐Resolution Imaging of Mitochondria

Macrophages were fixed in 4% PFA for 15 min and permeabilized with 0.25% Triton X‐100 for 5 min. After three PBS washes, cells were blocked with 5% BSA for 2 h at room temperature, followed by overnight incubation at 4 °C with a primary antibody against TOM20 (CAS no. sc‐17764, Santa Cruz, 1:200). After additional PBS washes, cells were incubated with a STAR RED–conjugated secondary antibody (CAS no. 41699, Abberior, 1:150) for 1 h at room temperature. Super‐resolution imaging was performed using a STED microscope (Abberior Instruments) equipped with a 100×oil‐immersion objective lens (NA 1.45, Olympus). The mitochondrial network was classified into three morphological categories: punctate, characterized by predominantly short and spherical mitochondria; filamentous, defined as networks in which more than 50% of mitochondria exceeded 3 µm in length and exhibited extensive interconnectivity; and intermediate, representing a mixed morphology that displayed features between the punctate and filamentous states. At least 11 cells per group were analyzed using Fiji software (version 2.1.0/1.53c).

### 5.12 Transmission Electron Microscopy

Macrophages were fixed in 2.5% glutaraldehyde and 1% paraformaldehyde in 0.1 M phosphate buffer (pH 7.4) for 1 h at room temperature. After rinsing in 0.1 M phosphate buffer, samples were post‐fixed in 1% osmium tetroxide containing 0.8% potassium ferricyanide (K4Fe(CN)6) for 1 h in the dark. Cells were subsequently rinsed and dehydrated through a graded acetone series (50%, 70%, 90%, 96%, and 100%; 5 min each). Dehydrated samples were embedded in Epon 812 resin and polymerized at 60°C for 48 h. Ultrathin sections (∼60 nm) were cut using a Leica UC7 ultramicrotome and mounted on 200‐mesh copper grids. Sections were stained with 5% uranyl acetate and lead citrate (10 min each) and examined using a Hitachi 7065B transmission electron microscope operated at 80 kV. Images were acquired with a Gatan SC1000 CCD camera. Mitochondria were identified by their characteristic double membrane and electron‐dense matrix, while LDs appeared as circular structures with a low‐contrast interior and less defined membranes. LD–mitochondrion contact was defined as direct physical apposition between an LD and the mitochondrial outer membrane. The frequency of LD–mitochondria contact was calculated as the percentage of mitochondria in direct contact with at least one LD. Mitochondrial morphology was quantified by a globularity index, defined as (minor axis/major axis) [2], with values closer to 1 indicating a more spherical shape. Morphometric analyses were performed in Fiji (version 2.1.0/1.53c) on multiple cells per condition.

### 5.13 Western Blot Analysis

Cells were washed with PBS and lysed in RIPA buffer (CAS no. P0013B, Beyotime) supplemented with 1 mM PMSF (CAS no. ST507, Beyotime) and a protease inhibitor cocktail (CAS no. P1005, Beyotime). Lysates were briefly sonicated (3–5 pulses) and incubated on ice for 30 min, followed by centrifugation at 12,000 g for 15 min at 4°C. Protein concentration in the supernatants was quantified using a BCA Protein Assay Kit (CAS no. A65453, ThermoFisher). Equal amounts of protein (20–30 µg) were mixed with SDS loading buffer and boiled for 10 min. Samples and molecular weight markers were separated on 10% SDS–PAGE gels and transferred to PVDF membranes (CAS no. IPVH00010, Merck). Membranes were blocked with 5% BSA in TBST (TBS + 0.1% Tween‐20) for 2 h at room temperature and incubated overnight at 4°C with the following primary antibodies: anti‐DRP1 (CAS no. ab184247, Abcam, 1:800), anti‐MFN1 (CAS no. 13196, Cell Signaling, 1:1000), anti‐MFN2 (CAS no. ab124773, Abcam, 1:500) and anti‐GAPDH (CAS no. AB‐P‐R001, 1:1000, Goodhere). After washing, membranes were incubated with appropriate HRP‐conjugated secondary antibodies for 1 h at room temperature. Signals were detected using enhanced chemiluminescence reagents and imaged with a Bio‐Rad ImageLab system.

### 5.14 SiRNA Transfection and Drug Treatment

For gene knockdown, macrophages were transfected with siRNAs targeting MFN1 and MFN2 (GenePharma) using Lipofectamine 3000 (CAS no. L3000001, ThermoFisher) according to the manufacturer's protocol. A non‐targeting siRNA (siCon) was used as a negative control. The siRNA sequences were as follows: siMFN1, 5′‐GCUAAACAGAUACUAGCUATT‐3′; siMFN2, 5′‐CCAGUAGUCCUCAAGGUUUTT‐3′; siCon, 5′‐UUCUCCGAACGUGUCACGUTT‐3′. Efficient knockdown of target proteins was confirmed by Western blotting.

For metabolic perturbation experiments, macrophages were pretreated with specific pharmacological inhibitors or vehicle (DMSO) as a control. Mitochondrial fission was inhibited using Mdivi‐1 (CAS no. M0199, Sigma, 25 µM, 1 h) [58]. FAO was inhibited using etomoxir (CAS no. 236020, Sigma, 200 µM, 3 h) [59]. Glycolysis was inhibited using 2DG (CAS no. D8375, Sigma, 10 mM, 1 h) [60].

### 5.15 SRS and TPEF Microscopy

SRS and TPEF imaging were performed using a picoEmerald laser system (Applied Physics & Electronics) operating at an 80 MHz repetition rate, similar to a previously described setup [34]. The system provides a synchronized picosecond pulse laser with a tunable pump beam (700–960 nm) and a fixed‐wavelength Stokes beam at 1031 nm. For SRS imaging, the Stokes beam was modulated at 20 MHz using an electro‐optic modulator. Transmission of the forward‐going pump and Stokes beams after passing through the samples was collected by a high numerical aperture (1.0) water condenser. A high optical density short‐pass filter (CAS no. ET980SP, Chroma) was used to block the Stokes beam completely and to transmit only the pump beam onto a large area silicon photodiode (CAS no. S3994‐01, Hamamatsu) with 48 DC reversed bias voltage. The signal was then extracted by a lock‐in amplifier (CAS no. HF2LI, Zurich Instruments). The analog output representing the SRS signal was fed into a data acquisition card (CAS no. PCIE‐6363, National Instruments) and input to the computer to display the image on LabVIEW 2018 software. To target specific vibrational modes, the pump beam was tuned to 796.8 nm (CH_2_, 2850 cm^−1^), 845 nm (C–D, 2107 cm^−1^ for PA‐d_31_), and 847 nm (C–D, 2135 cm^−1^ for glucose‐d_7_). The excitation power at the sample was approximately 20 mW for the pump beam and 90 mW for the Stokes beam across all SRS experiments.

For TPEF imaging, the pump beam was tuned to 796.8 nm, with an excitation power of approximately 40 mW at the sample. A photomultiplier tube (CAS no. H7422‐40, Hamamatsu) was used to detect the backward‐going TPEF signals through 520/40 nm (CAS no. ET520/40, Chroma) band‐pass filter.

All images were obtained in 400 × 400 pixels with a dwell time of 10 µs per pixel. No photodamage to cells was detected.

### 5.16 Quantification of Total and Newly Synthesized Lipids in LD

SRS image analysis was performed using ImageJ to quantify LD content. Total LDs were identified in CH_2_‐band images (2850 cm^−1^) by applying an intensity threshold to distinguish high‐signal regions from the cellular background. Newly synthesized lipids were detected in C–D‐band images at 2107 cm^−1^ (for PA‐d_31_) or 2135 cm^−1^ (for glucose‐d_7_) by similarly thresholding deuterium‐labeled lipid signals. Subsequently, by using the ImageJ “Analyze Particles” function, the LD abundance was measured based on the proportion of LD area to the total cell area. Then, LD area fraction can be quantified. The proportion of newly synthesized lipids was estimated by computing the ratio of C–D signal area to CH_2_ signal area.

### 5.17 Glucose Consumption Assay

Macrophages (non‐irradiated controls or cells irradiated at 625 or 850 nm) were cultured in fresh complete medium, with cell‐free medium incubated in parallel as a blank control. After 24 h, culture supernatants were collected and glucose concentrations were measured using a glucose assay kit (CAS no. BC2500, Solarbio) according to the manufacturer's instructions. Cells were then lysed, and total protein content was quantified using a BCA Protein Assay Kit (CAS no. A65453, ThermoFisher). Glucose consumption was calculated as the difference between glucose concentrations in the cell‐free control and each corresponding sample supernatant, and was normalized to total cellular protein content.

### 5.18 Glutamine Assay

Glutamine concentrations in culture supernatants were measured using a glutamine enzyme immunoassay kit (CAS no. TW210, TW‐REAGENG) according to the manufacturer's instructions. Macrophages were cultured in standard medium supplemented with 2 mM glutamine. After 24 h, culture supernatants were collected by centrifugation at 1000 × g for 5 min to remove debris. Optical density (OD) was measured using a Varioskan Flash Multimode Reader (ThermoFisher). In parallel, total protein content in corresponding cell lysates was determined using a BCA Protein Assay Kit. Glutamine uptake (%) was calculated as the percentage reduction in extracellular glutamine concentration relative to fresh medium and normalized to total cellular protein content.

### 5.19 GLS Activity Assay

GLS activity in macrophages was assessed using a GLS Activity Assay Kit (CAS no. BC1450, Solarbio), following the manufacturer's instructions. Cells were lysed by sonication in the supplied extraction buffer (on ice, total duration: 3 min; 3 s on/7 s off pulse cycle). The lysates were centrifuged at 12 000 × g for 15 min at 4°C to remove debris. Supernatants were then incubated with the assay reaction mixture for the indicated time. Absorbance was measured at 630 nm using a microplate reader against a reagent blank. GLS activity was calculated based on a standard calibration curve and normalized to total protein content in each sample.

### 5.20 Seahorse OCR and ECAR Measurements

Macrophages were seeded at a density of 1 × 10^4^ cells per well in XF96 microplates and subjected to PBM treatment as described above. OCR and ECAR were subsequently measured using a Seahorse XF96 Extracellular Flux Analyzer (Agilent Technologies). For OCR measurements, cells were sequentially injected with oligomycin (CAS no. 495455, Sigma, 1 µM; ATP synthase inhibitor), FCCP (CAS no. C2920, Sigma, 0.5 µM; mitochondrial uncoupler), and a combination of antimycin A (Sigma, A8674, 1 µM) and rotenone (CAS no. R8875, Sigma, 1 µM), which inhibit complexes III and I, respectively. These perturbations enabled calculation of basal respiration, ATP‐linked respiration (oligomycin‐sensitive), maximal respiration (FCCP‐stimulated), and non‐mitochondrial respiration (residual OCR after electron transport chain inhibition). For ECAR measurements, cells were sequentially injected with glucose (CAS no. 103577‐100, Agilent, 10 mM) to assess basal glycolysis, oligomycin (1 µM) to evaluate maximal glycolytic capacity by inhibiting oxidative phosphorylation, and 2‐DG (100 mM) to inhibit glycolysis and define non‐glycolytic acidification. Glycolytic parameters (basal ECAR, glycolytic capacity, and glycolytic reserve) were calculated from these measurements. Following each assay, OCR and ECAR values were normalized to protein content per well and analyzed according to the manufacturer's instructions.

### 5.21 Lactate Assay

The culture medium was harvested to assess lactate secretion from the cells. Lactate levels were measured using a colorimetric lactate assay kit (CAS no. D799851‐0050, Sangon) according to the manufacturer's instructions. Absorbance was recorded at 450 nm using a BioTek microplate reader. Lactate concentrations were normalized to total protein content quantified from the same samples.

### 5.22 ATP Production Assay

Intracellular ATP production was measured using an enhanced ATP assay kit (CAS no. S0026, Beyotime). Adhering strictly to the manufacturer's protocol, cells were lysed in lysis buffer on ice. Lysates were centrifuged at 12 000 g for 5 min at 4°C to remove debris. Supernatants were quickly added to the luciferase reaction solution from the kit, and luminescence was measured on a Tecan Spark luminometer. ATP concentrations were calculated from an ATP standard curve run in parallel and normalized to total protein in each sample.

### 5.23 In Vivo Wound‐Healing Model

The effects of PBM on wound healing were evaluated in vivo using healthy male BALB/c mice (20 g, 5 weeks old; Peking University Third Hospital). All experimental procedures were approved by the Biological and Medical Ethics Committee of Beihang University (approval number: BM20180011) and conducted in accordance with institutional guidelines for the Care and Use of Laboratory Animals. Mice were randomly assigned (*n* = 5 per group) to an untreated control group (Con) or to PBM treatment groups receiving irradiation at 625 or 850 nm at energy density of 10, 30, or 50 J/cm^2^. Sample size was estimated a priori using G*Power 3.1 to provide a pragmatic rationale for the in vivo wound‐healing experiment, based on preliminary quantitative data from H&E‐stained wound sections. Pilot estimates of the mean difference and within‐group variability in H&E‐based dermal gap length were used for the calculation, with α = 0.05 and power = 0.80. This analysis indicated that *n* = 5 animals per group was sufficient to detect the expected histological effect. Under sterile conditions, full‐thickness excisional wounds (10 mm diameter) were created on the dorsal skin using a sterile biopsy punch. Mice were anesthetized with sodium pentobarbital during wound creation. PBM treatment was applied once daily from Day 0 (the day of injury) through Day 12. During irradiation, the light source was positioned at a fixed distance of 1 cm from the wound surface, and animals were gently restrained to minimize movement and ensure a stable and reproducible irradiation geometry throughout treatment. Digital images of the wound sites were acquired on Days 4, 8, and 12 post‐injury. Wound closure was quantified using Fiji software, and the percentage of wound healing was calculated as: Healed area (%) = [(A_0_ – A_t_) / A_0_] × 100%, where A_0_ represents the initial wound area and A_t_ represents the wound area at Days 4, 8, or 12. On Day 12, mice were euthanized, and wound tissues were collected for histological analysis, including H&E staining, Masson's trichrome staining, evaluation of neovascularization, and assessment of macrophage polarization. Throughout the study, animals were housed under standard laboratory conditions: ambient temperature of 23 ± 2°C, relative humidity of 55 ± 5%, and a 12 h light–dark cycle. Mice had ad libitum access to tap water and standard chow, and bedding was changed three times per week.

### 5.24 Statistical Analysis

Data are presented as mean ± standard deviation (SD). Data from at least three independent biological experiments were analyzed using SPSS version 26.0 and GraphPad Prism version 9.0. Independent biological experiments were used as the statistical unit for all in vitro analyses. For in vivo analyses, individual animals were used as the statistical unit. Normality was assessed using the Shapiro–Wilk test. For comparisons among three or more groups, normally distributed data were analyzed using one‐way ANOVA. Homogeneity of variance was assessed using the Brown–Forsythe and Bartlett's tests. When the assumption of equal variances was met, post‐hoc comparisons were performed using Tukey's multiple‐comparisons test; when variances were unequal, Dunnett's T3 multiple‐comparisons test was used. Non‐normally distributed data involving three or more groups were analyzed using the Kruskal–Wallis test followed by Dunn's multiple‐comparisons test. For two‐group comparisons, normally distributed data with equal variances were analyzed using an unpaired two‐sided Student's *t*‐test, whereas normally distributed data with unequal variances were analyzed using Welch's *t*‐test. Non‐normally distributed two‐group data were analyzed using the Mann–Whitney U test. In the in vivo wound‐healing experiments, wound area and wound‐closure percentage were measured repeatedly in the same animals over time and analyzed using two‐way repeated‐measures ANOVA, with treatment group and time as factors and individual animals defined as the repeated‐measures unit. Post‐hoc multiple‐comparisons tests were performed to compare treatment groups at each time point. Effect sizes with 95% confidence intervals were calculated using independent biological experiment‐level values and are summarized in Table S6. Eta‐squared (η^2^) was used for one‐way ANOVA analyses, epsilon‐squared (ε^2^) for Kruskal–Wallis analyses, and Cohen's *d* for two‐group comparisons. No data were excluded from the analyses. A value of *p* < 0.05 was considered statistically significant.

## Author Contributions

L.Z., S.Y., and Y.F. conceived the project. L.Z., S.Y., and Q.S. designed the experiments. J.D. developed the custom‐built light irradiation device. Q.S. and H.J. performed the experiments. Q.S., H.J., L.L., J.C., X.C., Z.J., J.N., Z.Y., and X.C. analyzed the data. L.Z., S.Y., Q.S., H.J., and J.D. wrote the original and revised versions of the manuscript. All authors reviewed and approved the final manuscript.

## Conflicts of Interest

The authors declare no conflict of interest.

## Supporting information




**Supporting File**: advs75920‐sup‐0001‐SuppMat.docx.

## Data Availability

The data supporting the conclusions of this study are available in the Article and its Supplementary Information.
