# Supplementary material for: Wavelength‐Dependent Photobiomodulation Regulates Macrophage Polarization via Mitochondrial Dynamics and Metabolic Reprogramming
Source: Adv Sci (Weinh). 2026 Jun 2;13(48):e75920. doi: 10.1002/advs.75920 (PMC13336922; doi:10.1002/advs.75920)
Supplement: Supplementary file 1 [file ADVS-13-e75920-s001.docx]

**Supplementary materials**

**Wavelength-Dependent Photobiomodulation Regulates Macrophage Polarization via Mitochondrial Dynamics and Metabolic Reprogramming**

Qiusheng Shi^1#^, Hao Jia^1#^, Jianfei Dong^2#^, Linhao Li^1^, Jingqi Cao^1^, Xun Chen^1^, Zhenzhen Jia^1^, Jing Na^1^, Zhijie Yang^1^, Xinyuan Chen^1^, Yubo Fan^1*^, Shuhua Yue^1*^, Lisha Zheng^1*^

^1^Key Laboratory of Biomechanics and Mechanobiology (Beihang University), Ministry of Education, Beijing Advanced Innovation Center for Biomedical Engineering, School of Biological Science and Medical Engineering, Beihang University, Beijing, 100083, China.

^2^School of Future Science and Engineering, Soochow University, 1 Jiuyong West Road, Suzhou 215222, China.

**Correspondence**

*Yubo Fan, Shuhua Yue, and Lisha Zheng, Key Laboratory of Biomechanics and Mechanobiology (Beihang University), Ministry of Education, Beijing Advanced Innovation Center for Biomedical Engineering, School of Biological Science and Medical Engineering, Beihang University, No. 37 Xueyuan Road, Haidian District, Beijing, 100083, China.

**Email:** yubofan@buaa.edu.cn; yue_shuhua@buaa.edu.cn; lishazheng@buaa.edu.cn.

**This Word file includes:**

Supplementary Text

Figs. S1 to S16

Tabs. S1 to S6

**Fig. S1 Characterization and performance validation of the custom-built PBM irradiation system.** (A) Photograph of the complete PBM irradiation setup, including the control computer and software interface, incubator, CO_2_ supply, and system connections. (B) Emission spectra of the 625-, 850-, and 935-nm LED modules measured using a Maya2000Pro spectrometer. (C) Closed-loop irradiance control performance during 625-, 850-, and 935-nm illumination. Measured irradiance was monitored over time and plotted against the reference setpoint; shaded regions indicate the ±5% tolerance range around the target irradiance. (D) Irradiance distribution map showing spatially uniform illumination across a 5 × 5 cm irradiation field.

**Fig. S2 Cell viability after PBM at different wavelengths and energy density.** Cell viability was assessed by CCK-8 assay after irradiation at 625, 850, or 935 nm across increasing energy density (n = 4 independent biological experiments). Data are presented as mean ± s.d. Exact *p* values are shown in the figure.

**Fig. S3 Wavelength-dependent effects of PBM on macrophage polarization in mouse BMDMs.** (A) qRT–PCR analysis of M2-associated genes (*CD206*, *ARG-1*, *IL-10*) and M1-associated genes (*CCR7*, *TNF-α*, *IL-1β*) in BMDMs after irradiation at 625, 850, or 935 nm at 10, 30, or 50 J/cm^2^ (n = 4 independent biological experiments). (B) Immunofluorescence staining for CD206 (red) and CCR7 (green) under the conditions in (A), with representative images and quantification of the CD206/CCR7 fluorescence intensity ratio (scale bar, 50 µm; n = 4 independent biological experiments). (C) ELISA quantification of IL-10 and PDGF-BB in culture supernatants 24 h after irradiation at 625, 850, or 935 nm at the indicated doses (n = 3 independent biological experiments). Data are presented as mean ± s.d. Exact *p* values or significance levels are indicated in the figure. **p* < 0.05 and ^***^*p* < 0.01.

**Fig. S4 Induction and characterization of polarized macrophage phenotypes in THP-1–derived cells.** (A) Schematic of the differentiation and polarization workflow. THP-1 monocytes were differentiated into macrophages (M0) using PMA for 48 h, then polarized to M1 macrophages with LPS + IFN-γ or to M2 macrophages with IL-4 + IL-10 for an additional 48 h. (B) qRT–PCR analysis of M1-associated genes (*CCR7*, *TNF-α*, *IL-1B*) and M2-associated genes (*CD206*, *CD163*, *IL-10*) in M0, M1, and M2 macrophages (n = 4 independent biological experiments). (C) Immunofluorescence staining for CD206 (red) and CCR7 (green) in M0, M1, and M2 macrophages, with quantification of the CD206/CCR7 fluorescence intensity ratio (scale bar, 50 µm; n = 4 independent biological experiments). (D) ELISA quantification of PDGF-BB (M2-associated) and TNF-α (M1-associated) in culture supernatants from M0, M1, and M2 macrophages (n = 4 independent biological experiments). Data are presented as mean ± s.d. Exact *p* values or significance levels are indicated in the figure. ^*^*p* < 0.05 and ^***^*p* < 0.01.

**Fig. S5 Temperature-matched controls confirm that PBM-induced polarization is not driven by heating.** (A, B) Time course of culture medium temperature during PBM at 625, 850, or 935 nm at 10, 30, or 50 J/cm^2^, compared with non-irradiated controls (n = 4 independent biological experiments). (C) qRT–PCR analysis of M1- and M2-associated genes in THP-1–derived macrophages and mouse BMDMs incubated at 37.5, 38.0, or 38.5 ℃, showing no significant differences relative to 37.0 °C controls (n = 4 independent biological experiments). (D) ELISA quantification of cytokines in culture supernatants 24 h after incubation at the indicated temperatures. PDGF-BB and TNF-α were measured in THP-1–derived macrophages, and IL-10 and TNF-α in mouse BMDMs (n = 4 independent biological experiments). Data are presented as mean ± s.d. Exact *p* values or significance levels are indicated in the figure. ns not significant.

**Fig. S6 PBM-treated macrophages differentially regulate fibroblast migration and proliferation in Transwell co-culture.** (A) Representative images of fibroblast migration in a Transwell co-culture assay. Macrophages were irradiated at 625, 850, or 935 nm at 10, 30, or 50 J/cm^2^, and fibroblast migration was assessed 24 h later by crystal violet staining (scale bar, 100 µm). (B) Representative images from the EdU incorporation assay assessing fibroblast proliferation in Transwell co-culture under the conditions in (A). EdU-positive nuclei are shown in green, and total nuclei are counterstained with DAPI (blue) (scale bar, 100 µm).

**Fig. S7 Validation of MFN1 and MFN2 knockdown efficiency in macrophages.** (A) Western blot analysis of macrophages transfected with control siRNA or MFN1-specific siRNA, probed with an anti-MFN1 antibody to assess knockdown efficiency (n = 3 independent biological experiments). (B) Western blot analysis of macrophages transfected with control siRNA or MFN2-specific siRNA, probed with an anti-MFN2 antibody to assess knockdown efficiency (n = 3 independent biological experiments). Data are presented as mean ± s.d. Exact *p* values are shown in the figure.

**Fig. S8 MFN2 knockdown attenuates 850-nm–induced M2 polarization.** (A) qRT–PCR analysis of M2-associated genes (*CD206*, *CD163*, *IL10*) and M1-associated genes (*CCR7*, *TNF-α*, *IL-1β*) in macrophages transfected with control siRNA (siCon) or MFN2-specific siRNA (siMFN2) and subsequently irradiated with 850-nm light (50 J/cm^2^) (n = 5 independent biological experiments). (B) Immunofluorescence staining for CD206 (red) and CCR7 (green) under the conditions in (A), with quantification of the CD206/CCR7 fluorescence intensity ratio (scale bar, 50 µm; n = 4 independent biological experiments). (C) ELISA quantification of TNF-α and PDGF-BB in culture supernatants 24 h after 850-nm irradiation in siCon- or siMFN2-transfected macrophages (n = 4 independent biological experiments). Data are presented as mean ± s.d. Exact *p* values or significance levels are indicated in the figure. ^*^*p* < 0.05 and ^***^*p* < 0.01; ns not significant.

**Fig. S9 DRP1 inhibition preserves mitochondrial morphology in macrophages exposed to 625-nm red light.** Macrophages were treated with DMSO (control) or the DRP1 inhibitor Mdivi-1 (25 µM, 1 h), labeled with MitoTracker Red, and then irradiated with 625-nm red light (50 J/cm^2^). Mitochondrial morphology was quantified by mitochondrial branch length (scale bar, 2 µm; n = 4 independent biological experiments). Data are presented as mean ± s.d. Exact *p* values are shown in the figure.

**Fig. S10 850-nm NIR irradiation has minimal effects on *de novo* lipogenesis in macrophages.** Representative SRS images acquired at the CH_2_ band (~2850 cm^–1^) and C–D band (~2107 cm^–1^) in macrophages incubated with deuterium-labeled glucose (glucose-d_7_; 50 µM, 24 h) and subsequently irradiated with 850-nm NIR light (50 J/cm^2^) (scale bar, 20 µm). Quantification of LD content and the C–D/CH_2_ (CD_L_/CH_L_) ratio is shown (n = 4 independent biological experiments). Data are presented as mean ± s.d. Exact *p* values are shown in the figure. ns not significant.

**Fig. S11 850-nm NIR irradiation increases CPT1A protein expression in macrophages.** Representative western blot images and quantification of CPT1A protein expression in macrophages after irradiation with 850-nm NIR light (50 J/cm^2^). GAPDH was used as the loading control, and relative CPT1A protein levels were normalized to GAPDH (n = 3 independent biological experiments). Data are presented as mean ± s.d. Exact *p* values are shown in the figure.

**Fig. S12 Glucose uptake and glycolysis-related gene expression after 850-nm NIR irradiation.** (A) Glucose consumption in macrophages irradiated with 850-nm NIR light (50 J/cm^2^), normalized to total protein content (n = 4 independent biological experiments). (B) qRT–PCR analysis of glycolysis-related genes (*GLUT1*, *HK2*, *PFKM*, *LDHA*) under the conditions in (A) (n = 4 independent biological experiments). Data are presented as mean ± s.d. Exact *p* values are shown in the figure. ns not significant.

**Fig. S13 Glutamine uptake and GLS activity after 850-nm NIR irradiation.** (A) Glutamine uptake in macrophages irradiated with 850-nm NIR light (50 J/cm^2^), normalized to total protein content (n = 4 independent biological experiments). (B) GLS activity in macrophages under the conditions in (A), normalized to total protein content (n = 4 independent biological experiments). Data are presented as mean ± s.d. Exact *p* values are shown in the figure. ns not significant.

**Fig. S14 625-nm red-light irradiation increases HK2 protein expression in macrophages.** Representative western blot images and quantification of HK2 protein expression in macrophages after 625-nm irradiation at 50 J/cm^2^, with GAPDH used as the loading control. Relative HK2 protein levels were normalized to GAPDH (n = 3 independent biological experiments). Data are presented as mean ± s.d. Exact *p* values are shown in the figure.

**Fig. S15 Effects of 625-nm red-light irradiation on fatty acid uptake and *de novo* lipogenesis in macrophages.** (A) Representative SRS images acquired at the CH_2_ band (~2850 cm^–1^) and C–D band (~2107 cm^–1^) in macrophages incubated with deuterium-labeled palmitic acid (PA-d_31_; 25 µM, 24 h) and subsequently irradiated with 625-nm red light (50 J/cm^2^) (scale bar, 20 µm). Quantification of LD content and the C–D/CH_2_ (CD_L_/CH_L_) ratio is shown (n = 4 independent biological experiments). (B) Representative SRS images acquired at the CH₂ band (~2850 cm^–1^) and C–D band (~2107 cm^–1^) in macrophages incubated with deuterium-labeled glucose (glucose-d_7_; 50 µM, 24 h) and subsequently irradiated with 625-nm red light (50 J/cm^2^) (scale bar, 20 µm). Quantification of LD content and the C–D/CH_2_ (CD_L_/CH_L_) ratio is shown (n = 4 independent biological experiments). Data are presented as mean ± s.d. Exact *p* values are shown in the figure. ns not significant.

**Fig. S16 Glutamine uptake and GLS activity after 625-nm red-light irradiation.** (A) Glutamine uptake in macrophages irradiated with 625-nm red light (50 J/cm^2^), normalized to total protein content (n = 4 independent biological experiments). (B) GLS activity in macrophages under the conditions in (A), normalized to total protein content n = 4 independent biological experiments). Data are presented as mean ± s.d. Exact *p* values are shown in the figure. ns not significant.

**Tables**

**Tab. S1** **Detailed dosimetry parameters for PBM irradiation conditions (625, 850, and 935 nm).**

| Wavelength | Irradiance | Dose | Light spot size |
| --- | --- | --- | --- |
| 625 nm | 50 mW/cm^2^ | 10, 30, 50, 70, 90 J/cm^2^ | 5 cm × 5 cm |
| 850 nm | 50 mW/cm^2^ | 10, 30, 50, 70, 90 J/cm^2^ | 5 cm × 5 cm |
| 935 nm | 50 mW/cm^2^ | 10, 30, 50, 70, 90 J/cm^2^ | 5 cm × 5 cm |

**Tab. S2** **Comparison of fibroblast migration in Transwell co-culture with macrophages irradiated at 850 or 935 nm (10, 30, and 50 J/cm^2^).**

| Group | Migration of co-cultured fibroblasts in response to macrophages irradiated at 850 or 935 nm (mean ± s.d.^a^) | | *p* value^b^ |
| --- | --- | --- | --- |
|  | 850 nm | 935 nm |  |
| 10 J/cm^2^ | 123.485 ± 13.780 | 104.546 ± 13.682 | < 0.001^***^ |
| 30 J/cm^2^ | 186.273 ± 11.946 | 110.515 ± 16.722 | < 0.001^***^ |
| 50 J/cm^2^ | 194.667 ± 16.174 | 180.121 ± 17.161 | < 0.001^***^ |

^a^s.d. = standard deviation

^b^Migration was compared between 850- and 935-nm conditions at the same energy density using a two-sided unpaired Student’s *t*-test. Statistical significance was defined as ^*^*p* < 0.05 and ^***^*p* < 0.01.

**Tab. S3 Comparison of fibroblast proliferation in Transwell co-culture with macrophages irradiated at 850 or 935 nm (10, 30, and 50 J/cm^2^).**

| Group | Proliferation of co-cultured fibroblasts in response to macrophages irradiated at 850 or 935 nm (mean ± s.d.^a^) | | *p* value^b^ |
| --- | --- | --- | --- |
|  | 850 nm | 935 nm |  |
| 10 J/cm^2^ | 36.378 ± 4.692 | 39.433 ± 5.165 | = 0.0362^*^ |
| 30 J/cm^2^ | 52.891 ± 6.234 | 48.594 ± 7.524 | = 0.0359^*^ |
| 50 J/cm^2^ | 56.022 ± 6.054 | 52.504 ± 6.523 | = 0.0574 |

^a^s.d. = standard deviation

^b^Proliferation was compared between 850- and 935-nm conditions at the same energy density using a two-sided unpaired Student’s *t*-test. Statistical significance was defined as **p* < 0.05 and ****p* < 0.01.

**Tab. S4** **Comparison of *α-SMA*, *COL1A1*, and *VEGF* gene expression in fibroblasts co-cultured with macrophages irradiated at 850 or 935 nm (10, 30, and 50 J/cm^2^)**

| Group | Gene symbol | Gene expression in fibroblasts co-cultured with macrophages irradiated at 850 or 935 nm (Mean ±s.d.^a^) | | *p* value^b^ |
| --- | --- | --- | --- | --- |
|  |  | 850 nm | 935 nm |  |
| 10 J/cm^2^ | *α-SMA* | 1.585 ± 0.165 | 1.066 ± 0.284 | = 0.0146^*^ |
|  | *COL1A1* | 1.121 ± 0.207 | 0.999 ± 0.127 | = 0.2907 |
|  | *VEGF* | 1.280 ± 0.193 | 1.035 ± 0.152 | = 0.0564 |
|  |  |  |  |  |
| 30 J/cm^2^ | *α-SMA* | 1.968 ± 0.242 | 1.560 ± 0.162 | = 0.0189^*^ |
|  | *COL1A1* | 1.568 ± 0.149 | 1.470 ± 0.171 | = 0.3639 |
|  | *VEGF* | 1.568 ± 0.172 | 0.964 ± 0.148 | = 0.0003^***^ |
|  |  |  |  |  |
| 50 J/cm^2^ | *α-SMA* | 2.181 ± 0.423 | 1.872 ± 0.246 | = 0.2104 |
|  | *COL1A1* | 1.765 ± 0.160 | 1.557 ± 0.175 | = 0.0864 |
|  | *VEGF* | 1.561 ± 0.242 | 1.545 ± 0.071 | = 0.8899 |

^a^s.d. = standard deviation

^b^Gene expression was compared between 850- and 935-nm conditions at the same energy density using a two-sided unpaired Student’s *t*-test. Statistical significance was defined as **p* < 0.05 and ****p* < 0.01.

**Tab. S5 Primer sequences used for qRT–PCR.**

| Gene symbol | Species | ID gene | Sequence (5’ - 3’) |
| --- | --- | --- | --- |
| *CD206* | Human | NM_002438 | F: GGACGTGGCTGTGGATAAAT |
|  |  |  | R: ACCCAGAAGACGCATGTAAAG |
| *CD163* | Human | NM_004244 | F: ATCAACCCTGCATCTTTAGACA |
|  |  |  | R: CTTGTTGTC ACATGTGATCCAG |
| *IL-10* | Human | NM_000572 | F: CTTTAAGGGTTACCTGGGTTGC |
|  |  |  | R: CCTTGATGTCTGGGTCTTGGT |
| *CCR7* | Human | NM_001301717 | F: TGAGGTCACGGACGATTACAT |
|  |  |  | R: GTAGGCCCACGAAACAAATGAT |
| *TNF-α* | Human | NM_000594 | F: CTCCTCACCCACACCATCAGCCGCA |
|  |  |  | R: ATAGATGGGCTCATACCAGGGCTTG |
| *IL-1β* | Human | NM_000576 | F: GCCAGTGAAATGATGGCTTATT |
|  |  |  | R: AGGAGCACTTCATCTGTTTAGG |
| *α-SMA* | Human | NM_001406462 | F: CCACCCTGCTCACGGAGGCAC |
|  |  |  | R: CATAGTGGTGCCCCCTGATAG |
| *COL1A1* | Human | NM_000088 | F: GTCGAGGGCCAAGACGAA |
|  |  |  | R: GTCTCGGTCATGGTACCT |
| *VEGF* | Human | NM_001025366 | F: GAGATGAGCTTCCTACAGCAC |
|  |  |  | R: TCACCGCCTCGGCTTGTCACAT |
| *ACSL1* | Human | NM_001995 | F: ACACACCTGACAAGGCCAAA |
|  |  |  | R: GGGTTTCCTGTCGTTCCACT |
| *CPT1A* | Human | NM_004377 | F: GGTGAACAGCAACTATTATGTC |
|  |  |  | R: ATCCTCTGGAACTGCATC |
| *CPT1B* | Human | NM_198839 | F: TTCATGGGTCCTCCAAGCCA |
|  |  |  | R: TTTTCCTGCCAGTCCACACG |
| *CPT2* | Human | NM_004035 | F: GCCCAGGTGAAGCCTGATGGA |
|  |  |  | R: GACTGGTGCCTCACAGCGCTG |
| *NDUFB8* | Human | NM_001199984 | F: TTAGCAAATCACCCATTGGACTG |
|  |  |  | R: CCCCTCTAAAAATCGGCTCCTA |
| *UQCRC2* | Human | NM_001294332 | F: TCGCACTGTGCATAGAGGAC |
|  |  |  | R: ATGCCTGTAGGGTGGAACTG |
| *SDHB* | Human | NM_003365 | F: TGTCTCGTGCAGACTTGACC |
|  |  |  | R: GGCGAGGTCTAACAGTTGCT |
| *ATP5F1B* | Human | NM_001257335 | F: ATGACGACTTATCCAAACAGGC |
|  |  |  | R: CGGGAGTGTAGGTAGAACACAT |
| *GLUT1* | Human | NM_001371525 | F: GCCATCCTGCAACACTTAGGGCTTGAG |
|  |  |  | R: GTGAGGATGTAGCTTGTAGAGGGTCCC |
| *HK2* | Human | NM_002654 | F: ATGACCCATGAAGAGCACCA |
|  |  |  | R: GCACCGGTGAAGATACCAAC |
| *PFKM* | Human | NM_001166687 | F: CGCATGCAGCACCTGATTG |
|  |  |  | R: GCGGCGGAGTTCCTCAAATA |
| *LDHA* | Human | NM_001165416 | F: GATTCAGCCCGATTCCGTTA |
|  |  |  | R: CATACAGGCACACTGGAATCT |
| *ACTB* | Human | NM_001101 | F: GGCACCCAGCACAATGAAG |
|  |  |  | R: CCGATCCACACGGAGTACTTG |
| *CD206* | Mouse | NM_008625 | F: CTCTGTTCAGCTATTGGACGC |
|  |  |  | R: CGGAATTTCTGGGATTCAGCTTC |
| *ARG1* | Mouse | NM_007482 | F: TTTTTCCAGCAGACCAGCTT |
|  |  |  | R: AGAGATTATCGGAGCGCCTT |
| *IL-10* | Mouse | NM_010548 | F: GCTCTTACTGACTGGCATGAG |
|  |  |  | R: CGCAGCTCTAGGAGCATGTG |
| *CCR7* | Mouse | NM_007719 | F: TGTACGAGTCGGTGTGCTTC |
|  |  |  | R: GGTAGGTATCCGTCATGGTCTTG |
| *TNF-α* | Mouse | NM_013693 | F: ATGAGCACAGAAAGCATGATCCGC |
|  |  |  | R: AAAGTAGACCTGCCCGGACTC |
| *IL-1β* | Mouse | NM_008361 | F: GCAACTGTTCCTGAACTCAACT |
|  |  |  | R: ATCTTTTGGGGTCCGTCAACT |
| *ACTB* | Mouse | NM_007393 | F: GGCTGTATTCCCCTCCATCG |
|  |  |  | R: CCAGTTGGTAACAATGCCATGT |

**Tab. S6 Effect-size estimates and 95% confidence intervals for the presented datasets.**

| Data | Endpoint | | Effect size metric | Effect size value | 95% confidence intervals |
| --- | --- | --- | --- | --- | --- |
| Fig. 1D | 625 nm | *CD206* | η^2^ | 0.8286 | [0.4675, 0.8845] |
|  |  | *CD163* | η^2^ | 0.8741 | [0.5894, 0.9148] |
|  |  | *IL-10* | η^2^ | 0.8010 | [0.2232, 0.8674] |
|  |  | *CCR7* | η^2^ | 0.9065 | [0.6854, 0.9366] |
|  |  | *TNF-α* | η^2^ | 0.9309 | [0.7621, 0.9530] |
|  |  | *IL-1β* | η^2^ | 0.7649 | [0.4175, 0.8395] |
|  | 850 nm | *CD206* | η^2^ | 0.9652 | [0.8768, 0.9762] |
|  |  | *CD163* | η^2^ | 0.9406 | [0.7786, 0.9597] |
|  |  | *IL-10* | η^2^ | 0.8611 | [0.6290, 0.9048] |
|  |  | *CCR7* | η^2^ | 0.8018 | [0.4025, 0.8666] |
|  |  | *TNF-α* | ε^2^ | 0.8163 | [0.4141, 0.8760] |
|  |  | *IL-1β* | η^2^ | 0.8614 | [0.5541, 0.9064] |
|  | 935 nm | *CD206* | η^2^ | 0.9083 | [0.6910, 0.9378] |
|  |  | *CD163* | η^2^ | 0.8269 | [0.4631, 0.8833] |
|  |  | *IL-10* | η^2^ | 0.8780 | [0.6007, 0.9174] |
|  |  | *CCR7* | η^2^ | 0.8407 | [0.4984, 0.8925] |
|  |  | *TNF-α* | η^2^ | 0.8160 | [0.4364, 0.8761] |
|  |  | *IL-1β* | η^2^ | 0.9175 | [0.7195, 0.9440] |
| Fig. 1E | 625 nm | CD206/CCR7 | η^2^ | 0.9320 | [0.7659, 0.9538] |
|  | 850 nm |  | η^2^ | 0.9806 | [0.9305, 0.9867] |
|  | 935 nm |  | η^2^ | 0.9427 | [0.8010, 0.9610] |
| Fig. 1F | 625 nm | PDGF-BB | ε^2^ | 0.9472 | [0.8140, 0.9640] |
|  |  | TNF-α | η^2^ | 0.9749 | [0.9105, 0.9829] |
|  | 850 nm | PDGF-BB | η^2^ | 0.9943 | [0.9794, 0.9961] |
|  |  | TNF-α | ε^2^ | 0.9439 | [0.8030, 0.9618] |
|  | 935 nm | PDGF-BB | η^2^ | 0.9916 | [0.9695, 0.9942] |
|  |  | TNF-α | η^2^ | 0.9747 | [0.9096, 0.9827] |
| Fig. 1H | 625 nm | Migration | η^2^ | 0.8578 | [0.3817, 0.9046] |
|  | 850 nm |  | η^2^ | 0.9815 | [0.9006, 0.9874] |
|  | 935 nm |  | η^2^ | 0.9828 | [0.9076, 0.9883] |
| Fig. 1I | 625 nm | Proliferation | η^2^ | 0.8116 | [0.2493, 0.8743] |
|  | 850 nm |  | η^2^ | 0.9640 | [0.8118, 0.9755] |
|  | 935 nm |  | η^2^ | 0.9634 | [0.8091, 0.9751] |
| Fig. 1J | 625 nm | α-SMA | η^2^ | 0.7956 | [0.4805, 0.8603] |
|  | 850 nm |  | η^2^ | 0.7798 | [0.3528, 0.8520] |
|  | 935 nm |  | η^2^ | 0.7644 | [0.4164, 0.8391] |
|  | 625 nm | VEGF | η^2^ | 0.7566 | [0.4500, 0.8310] |
|  | 850 nm |  | η^2^ | 0.6727 | [0.2541, 0.7766] |
|  | 935 nm |  | η^2^ | 0.5956 | [0.0900, 0.7239] |
|  | 625 nm | COL1A1 | η^2^ | 0.7363 | [0.4243, 0.8170] |
|  | 850 nm |  | η^2^ | 0.7745 | [0.4368, 0.8460] |
|  | 935 nm |  | η^2^ | 0.8004 | [0.4908, 0.8636] |
| Fig. 2F | 850 nm | Wound edge | Cohen’s *d* | 4.4980 | [1.9767, 6.9589] |
| Fig. 2G | 850 nm | Collagen | Cohen’s *d* | -5.0404 | [-7.7324, -2.2935] |
| Fig. 2H | 850 nm | α-SMA | Cohen’s *d* | -7.2001 | [-10.8530, -3.5125] |
| Fig. 2I | 850 nm | CD31 | Cohen’s *d* | -8.2233 | [-12.3446, -4.0751] |
|  |  | CD34 | Cohen’s *d* | -4.5996 | [-7.1033, -2.0365] |
| Fig. 2J | 850 nm | CD206 | Cohen’s *d* | -2.8743 | [-4.6937, -0.9795] |
|  |  | CCR7 | Cohen’s *d* | 5.1746 | [2.3711, 7.9247] |
| Fig. 2O | 625 nm | Wound edge | Cohen’s *d* | -5.6234 | [-8.5696, -2.6283] |
| Fig. 2P | 625 nm | Collagen | Cohen’s *d* | 4.4442 | [1.9449, 6.8825] |
| Fig. 2Q | 625 nm | α-SMA | Cohen’s *d* | 3.9424 | [1.6450, 6.1734] |
| Fig. 2R | 625 nm | *CD31* | Cohen’s *d* | 4.1142 | [1.7485, 6.4154] |
|  |  | *CD34* | Cohen’s *d* | 7.2825 | [3.5580, 10.9728] |
| Fig. 2S | 625 nm | *CD206* | Cohen’s *d* | 4.6729 | [2.0795, 7.2076] |
|  |  | *CCR7* | Cohen’s *d* | -3.1720 | [-5.1011, -1.1697] |
| Fig. 3A | 850 nm | Mitochondrial length | η^2^ | 0.7534 | [0.2977, 0.8344] |
| Fig. 3C | 850 nm | Sphericity | Cohen’s *d* | 3.0503 | [0.8249, 5.1849] |
| Fig. 3D | 850 nm | MFN1 | η^2^ | 0.8869 | [0.6264, 0.9234] |
|  |  | MFN2 | η^2^ | 0.9201 | [0.7277, 0.9457] |
|  |  | DRP1 | η^2^ | 0.8406 | [0.4983, 0.8925] |
| Fig. 3E | 625 nm | Mitochondrial length | η^2^ | 0.7829 | [0.3596, 0.8540] |
| Fig. 3G | 625 nm | Sphericity | Cohen’s *d* | -2.9946 | [-5.1054, -0.7924] |
| Fig. 3H | 625 nm | MFN1 | η^2^ | 0.2572 | [0.0000, 0.4714] |
|  |  | MFN2 | η^2^ | 0.9369 | [0.7818, 0.9571] |
|  |  | DRP1 | η^2^ | 0.9323 | [0.7668, 0.9540] |
| Fig. 3I | 850 nm | *CD206* | η^2^ | 0.9730 | [0.9227, 0.9813] |
|  |  | *CD163* | ε^2^ | 0.9239 | [0.7868, 0.9476] |
|  |  | *IL-10* | η^2^ | 0.9657 | [0.9024, 0.9763] |
|  |  | *CCR7* | ε^2^ | 0.9216 | [0.7805, 0.9460] |
|  |  | *TNF-α* | η^2^ | 0.9006 | [0.7273, 0.9317] |
|  |  | *IL-1β* | η^2^ | 0.8943 | [0.7111, 0.9274] |
| Fig. 3J | 850 nm | CD206/CCR7 | η^2^ | 0.9854 | [0.9475, 0.9900] |
| Fig. 3K | 850 nm | PDGF-BB | η^2^ | 0.9949 | [0.9815, 0.9965] |
|  |  | TNF-α | η^2^ | 0.9905 | [0.9659, 0.9935] |
| Fig. 3L | 625 nm | *CD206* | η^2^ | 0.9809 | [0.9526, 0.9866] |
|  |  | *CD163* | η^2^ | 0.9502 | [0.8775, 0.9651] |
|  |  | *IL-10* | η^2^ | 0.9775 | [0.9440, 0.9842] |
|  |  | *CCR7* | η^2^ | 0.9300 | [0.8294, 0.9511] |
|  |  | *TNF-α* | η^2^ | 0.9334 | [0.8375, 0.9535] |
|  |  | *IL-1β* | η^2^ | 0.9325 | [0.8353, 0.9529] |
| Fig. 3M | 625 nm | CD206/CCR7 | ε^2^ | 0.9730 | [0.9033, 0.9815] |
| Fig. 3N | 625 nm | PDGF-BB | η^2^ | 0.9792 | [0.9225, 0.9858] |
|  |  | TNF-α | ε^2^ | 0.9937 | [0.9773, 0.9957] |
| Fig. 4B | 850 nm | LD area | Cohen’s *d* | 2.9976 | [0.9159, 5.0054] |
|  |  | CD_L_/CH_L_ | Cohen’s *d* | 3.3587 | [1.0022, 5.6287] |
| Fig. 4D | 850 nm | Mitochondria -LDs | Cohen’s *d* | -3.7836 | [-6.2474, -1.2399] |
| Fig. 4E | 850 nm | *ACSL1* | Cohen’s *d* | -10.6513 | [-16.6708, -4.6462] |
|  |  | *CPT1A* | Cohen’s *d* | -6.3482 | [-10.0852, -2.5733] |
|  |  | *CPT1B* | Cohen’s *d* | -10.6145 | [-16.6142, -4.6288] |
|  |  | *CPT2* | Cohen’s *d* | -7.8042 | [-12.3026, -3.2878] |
|  |  | *NDUFB8* | Cohen’s *d* | -9.0537 | [-14.2160, -3.8886] |
|  |  | *UQCRC2* | Cohen’s *d* | -9.3020 | [-14.5970, -4.0070] |
|  |  | *SDHB* | Cohen’s *d* | -11.5000 | [-17.9775, -5.0452] |
|  |  | *ATP5F1B* | Cohen *d* | -10.0637 | [-15.7670, -4.3686] |
| Fig. 4G | 850 nm | Basal respiration | Cohen’s *d* | -3.3519 | [-5.1587, -1.4842] |
|  |  | ATP production | Cohen’s *d* | -2.6975 | [-4.2901, -1.0413] |
|  |  | Maximal respiration | Cohen’s *d* | -5.0626 | [-7.4978, -2.5785] |
|  |  | Spare respiratory capacity | Cohen’s *d* | -3.9343 | [-5.9463, -1.8650] |
| Fig. 4H | 850 nm | ATP levels | Cohen’s *d* | -16.7772 | [-28.0991, -5.6957] |
| Fig. 4I | 625 nm | Glucose consumption | Cohen’s *d* | -13.9285 | [-23.3640, -4.6756] |
| Fig. 4J | 625 nm | *GLUT1* | Cohen’s *d* | -8.2492 | [-12.3823, -4.0892] |
|  |  | *HK2* | Cohen’s *d* | -6.6564 | [-10.0630, -3.2104] |
|  |  | *PFKM* | Cohen’s *d* | -5.7825 | [-8.7988, -2.7188] |
|  |  | *LDHA* | Cohen’s *d* | -5.5378 | [-8.4463, -2.5795] |
|  |  | *NDUFB8* | Cohen’s *d* | 4.1392 | [1.9967, 6.2260] |
|  |  | *UQCRC2* | Cohen’s *d* | 3.8237 | [1.7935, 5.7960] |
|  |  | *SDHB* | Cohen’s *d* | 5.1169 | [2.6122, 7.5730] |
|  |  | *ATP5F1B* | Cohen’s *d* | 6.9080 | [3.7038, 10.0754] |
| Fig. 4L | 625 nm | Glycolysis | Cohen’s *d* | -4.4143 | [-6.6030, -2.1718] |
|  |  | Glycolytic capacity | Cohen’s *d* | -3.2225 | [-4.9853, -1.3980] |
|  |  | Glycolytic reserve | Cohen’s *d* | 0.3576 | [-0.7932, 1.4910] |
| Fig. 4M | 625 nm | Lactate level | Cohen’s *d* | -11.3112 | [-19.0221, -3.7260] |
| Fig. 4N | 625 nm | ATP levels | Cohen’s *d* | -6.0431 | [-10.3493, -1.7238] |
| Fig. 5A | 850 nm | *CD206* | η^2^ | 0.9899 | [0.9635, 0.9931] |
|  |  | *CD163* | η^2^ | 0.9744 | [0.9086, 0.9825] |
|  |  | *IL-10* | η^2^ | 0.9609 | [0.8621, 0.9733] |
|  |  | *CCR7* | η^2^ | 0.9852 | [0.9470, 0.9899] |
|  |  | *TNF-α* | η^2^ | 0.8978 | [0.6588, 0.9307] |
|  |  | *IL-1β* | η^2^ | 0.9618 | [0.8650, 0.9739] |
| Fig. 5B | 850 nm | CD206/CCR7 | η^2^ | 0.9943 | [0.9794, 0.9961] |
| Fig. 5C | 850 nm | PDGF-BB | η^2^ | 0.9828 | [0.9077, 0.9883] |
|  |  | TNF-α | η^2^ | 0.9730 | [0.8569, 0.9816] |
| Fig. 5D | 850 nm | Glucose consumption | η^2^ | 0.9869 | [0.9292, 0.9910] |
| Fig. 5E | 850 nm | *GLUT1* | η^2^ | 0.9556 | [0.8438, 0.9697] |
|  |  | *HK2* | η^2^ | 0.8579 | [0.5445, 0.9040] |
|  |  | *PFKM* | η^2^ | 0.9244 | [0.7413, 0.9486] |
|  |  | *PKM2* | ε^2^ | 0.9358 | [0.7758, 0.9563] |
| Fig. 5F | 850 nm | Lactate level | η^2^ | 0.9681 | [0.8321, 0.9783] |
| Fig. 5G | 850 nm | Sphericity | η^2^ | 0.8351 | [0.4841, 0.8888] |
| Fig. 5H | 625 nm | *CD206* | η^2^ | 0.9841 | [0.9429, 0.9891] |
|  |  | *CD163* | η^2^ | 0.9737 | [0.9064, 0.9821] |
|  |  | *IL-10* | η^2^ | 0.9556 | [0.8439, 0.9697] |
|  |  | *CCR7* | η^2^ | 0.9635 | [0.8710, 0.9751] |
|  |  | *TNF-α* | η^2^ | 0.9175 | [0.7194, 0.9439] |
|  |  | *IL-1β* | η^2^ | 0.9539 | [0.8383, 0.9686] |
| Fig. 5I | 625 nm | CD206/CCR7 | η^2^ | 0.9751 | [0.9110, 0.9830] |
| Fig. 5J | 625 nm | PDGF-BB | η^2^ | 0.9944 | [0.9692, 0.9961] |
|  |  | TNF-α | η^2^ | 0.9935 | [0.9646, 0.9956] |
| Fig. 5K | 625 nm | LD area | ε^2^ | 0.7491 | [0.0000, 0.8325] |
|  |  | CD_L_/CH_L_ | η^2^ | 0.9128 | [0.5816, 0.9411] |
| Fig. 5L | 625 nm | *ACSL1* | ε^2^ | 0.9183 | [0.7180, 0.9444] |
|  |  | *CPT1A* | η^2^ | 0.9145 | [0.7102, 0.9420] |
|  |  | *CPT1B* | η^2^ | 0.9768 | [0.9174, 0.9842] |
|  |  | *CPT2* | η^2^ | 0.9167 | [0.7169, 0.9434] |
|  |  | *NDUFB8* | η^2^ | 0.9080 | [0.6901, 0.9376] |
|  |  | *UQCRC2* | ε^2^ | 0.8592 | [0.5340, 0.9047] |
|  |  | *SDHB* | η^2^ | 0.9535 | [0.8370, 0.9683] |
|  |  | *ATP5F1B* | η^2^ | 0.9345 | [0.7739, 0.9554] |
| Fig. 5M | 625 nm | Sphericity | η^2^ | 0.8749 | [0.5918, 0.9154] |
| Fig. S2 | 625 nm | Viability | η^2^ | 0.9316 | [0.7355, 0.9477] |
|  | 850 nm |  | η^2^ | 0.9027 | [0.6339, 0.9258] |
|  | 935 nm |  | η^2^ | 0.9388 | [0.7619, 0.9532] |
| Fig. S3A | 625 nm | *CD206* | η^2^ | 0.8120 | [0.4267, 0.8734] |
|  |  | *ARG-1* | η^2^ | 0.7374 | [0.2666, 0.8237] |
|  |  | *IL-10* | η^2^ | 0.7937 | [0.3838, 0.8612] |
|  |  | *CCR7* | ε^2^ | 0.9421 | [0.7969, 0.9605] |
|  |  | *TNF-α* | η^2^ | 0.9408 | [0.7946, 0.9597] |
|  |  | *IL-1β* | η^2^ | 0.9250 | [0.7432, 0.9490] |
|  | 850 nm | *CD206* | η^2^ | 0.9593 | [0.8564, 0.9722] |
|  |  | *ARG-1* | η^2^ | 0.9270 | [0.7496, 0.9504] |
|  |  | *IL-10* | η^2^ | 0.8358 | [0.4858, 0.8892] |
|  |  | *CCR7* | η^2^ | 0.8894 | [0.6339, 0.9251] |
|  |  | *TNF-α* | ε^2^ | 0.7903 | [0.3459, 0.8586] |
|  |  | *IL-1β* | η^2^ | 0.8528 | [0.5305, 0.9006] |
|  | 935 nm | *CD206* | η^2^ | 0.9286 | [0.7549, 0.9515] |
|  |  | *ARG-1* | η^2^ | 0.7630 | [0.3172, 0.8408] |
|  |  | *IL-10* | η^2^ | 0.8785 | [0.6020, 0.9177] |
|  |  | *CCR7* | η^2^ | 0.7557 | [0.3025, 0.8360] |
|  |  | *TNF-α* | η^2^ | 0.7564 | [0.3039, 0.8364] |
|  |  | *IL-1β* | ε^2^ | 0.7449 | [0.2362, 0.8284] |
| Fig. S3B | 625 nm | CD206/CCR7 | η^2^ | 0.7917 | [0.3793, 0.8599] |
|  | 850 nm |  | η^2^ | 0.9755 | [0.9124, 0.9832] |
|  | 935 nm |  | η^2^ | 0.9288 | [0.7553, 0.9516] |
| Fig. S3C | 625 nm | *IL-10* | η^2^ | 0.8603 | [0.3897, 0.9063] |
|  |  | *TNF-α* | η^2^ | 0.9072 | [0.5589, 0.9374] |
|  | 850 nm | *IL-10* | η^2^ | 0.9810 | [0.8984, 0.9871] |
|  |  | *TNF-α* | η^2^ | 0.9855 | [0.9217, 0.9901] |
|  | 935 nm | *IL-10* | η^2^ | 0.9857 | [0.9230, 0.9903] |
|  |  | *TNF-α* | η^2^ | 0.9401 | [0.6987, 0.9594] |
| Fig. S4B | M1 | *CD206* | Cohen’s *d* | 4.2116 | [1.4730, 6.8776] |
|  |  | *CD163* | Cohen’s *d* | 5.4370 | [2.1140, 8.7079] |
|  |  | *IL-10* | Cohen’s *d* | 5.7351 | [2.2655, 9.1573] |
|  |  | *CCR7* | Cohen’s *d* | -11.0156 | [-17.2315, -4.8177] |
|  |  | *TNF-α* | Cohen’s *d* | -8.9298 | [-14.0260, -3.8294] |
|  |  | *IL-1β* | Cohen’s *d* | -5.4444 | [-8.7190, -2.1177] |
|  | M2 | *CD206* | Cohen’s *d* | -13.9201 | [-21.7106, -6.1742] |
|  |  | *CD163* | Cohen’s *d* | -5.9956 | [-9.5510, -2.3969] |
|  |  | *IL-10* | Cohen’s *d* | -6.8340 | [-10.8232, -2.8138] |
|  |  | *CCR7* | Cohen’s *d* | 7.0388 | [2.9146, 11.1350] |
|  |  | *TNF-α* | Cohen’s *d* | 12.7185 | [5.6150, 19.8560] |
|  |  | *IL-1β* | Cohen’s *d* | 5.4287 | [2.1097, 8.6954] |
| Fig. S4C | M1 | CD206/CCR7 | Cohen’s *d* | 6.8037 | [2.7989, 10.7770] |
|  | M2 |  | Cohen’s *d* | -6.5795 | [-10.4363, -2.6881] |
| Fig. S4D | M1 | PDGF-BB | Cohen’s *d* | 4.8422 | [1.8069, 7.8155] |
|  |  | TNF-α | Cohen’s *d* | -15.2957 | [-23.8357, -6.8119] |
|  | M2 | PDGF-BB | Cohen’s *d* | -18.0251 | [-28.0564, -8.0714] |
|  |  | TNF-α | Cohen’s *d* | 9.3197 | [4.0154, 14.6241] |
| Fig. S5C | Temperature (THP-1) | *CD206* | η^2^ | 0.0060 | [0.0000, 0.0060] |
|  |  | *CD163* | ε^2^ | 0.0431 | [0.0192, 0.6377] |
|  |  | *IL-10* | η^2^ | 0.0718 | [0.0000, 0.2362] |
|  |  | *CCR7* | η^2^ | 0.1214 | [0.0000, 0.3216] |
|  |  | *TNF-α* | η^2^ | 0.0353 | [0.0000, 0.1253] |
|  |  | *IL-1β* | η^2^ | 0.0440 | [0.0000, 0.1603] |
|  | Temperature (Mouse BMDMs) | *CD206* | η^2^ | 0.0546 | [0.0000, 0.1937] |
|  |  | *ARG-1* | η^2^ | 0.0952 | [0.0000, 0.2811] |
|  |  | *IL-10* | η^2^ | 0.1020 | [0.0000, 0.2937] |
|  |  | *CCR7* | η^2^ | 0.0971 | [0.0000, 0.2842] |
|  |  | *TNF-α* | η^2^ | 0.0038 | [0.0000, 0.0038] |
|  |  | *IL-1β* | η^2^ | 0.0337 | [0.0000, 0.1177] |
| Fig. S5D | Temperature (THP-1) | PDGF-BB | η^2^ | 0.0490 | [0.0000, 0.1769] |
|  |  | TNF-α | η^2^ | 0.0740 | [0.0000, 0.2409] |
|  | Temperature (Mouse BMDMs) | IL-10 | η^2^ | 0.1268 | [0.0000, 0.3293] |
|  |  | TNF-α | η^2^ | 0.1159 | [0.0000, 0.3137] |
| Fig. S7A | 850 nm | MFN1 | Cohen’s *d* | 6.4598 | [1.8909, 11.0285] |
| Fig. S7B | 850 nm | MFN2 | Cohen’s *d* | 10.8666 | [3.5629, 18.2857] |
| Fig. S8A | 850 nm | *CD206* | η^2^ | 0.9852 | [0.9574, 0.9897] |
|  |  | *CD163* | η^2^ | 0.9611 | [0.8893, 0.9731] |
|  |  | *IL-10* | η^2^ | 0.9331 | [0.8129, 0.9539] |
|  |  | *CCR7* | η^2^ | 0.9478 | [0.8527, 0.9640] |
|  |  | *TNF-α* | η^2^ | 0.8853 | [0.6886, 0.9213] |
|  |  | *IL-1β* | η^2^ | 0.8744 | [0.6612, 0.9138] |
| Fig. S8B | 850 nm | CD206/CCR7 | ε^2^ | 0.9724 | [0.9013, 0.9811] |
| Fig. S8C | 850 nm | PDGF-BB | η^2^ | 0.9828 | [0.9381, 0.9882] |
|  |  | TNF-α | η^2^ | 0.9861 | [0.9500, 0.9905] |
| Fig. S9 | 625 nm | Mitochondrial length | ε^2^ | 0.9472 | [0.8143, 0.9640] |
| Fig. S10 | 850 nm | LD area | Cohen’s *d* | -0.6364 | [-2.0421, 0.8177] |
|  |  | CD_L_/CH_L_ | Cohen’s *d* | 0.5353 | [-0.9026, 1.9317] |
| Fig. S11 | 850 nm | CPT1A | Cohen’s *d* | -8.4097 | [-14.2275, -2.6470] |
| Fig. S12A | 850 nm | Glucose consumption | Cohen’s *d* | -0.4038 | [-1.7916, 1.0160] |
| Fig. S12B | 850 nm | *GLUT1* | Cohen’s *d* | 0.2884 | [-1.1182, 1.6719] |
|  |  | *HK2* | Cohen’s *d* | -0.3593 | [-1.7451, 1.0550] |
|  |  | *PFKM* | Cohen’s *d* | 0.3360 | [-1.0757, 1.7209] |
|  |  | *LDHA* | Cohen’s *d* | 0.0554 | [-1.3330, 1.4394] |
| Fig. S13A | 850 nm | Glutamine uptake | Cohen’s *d* | -0.3595 | [-1.7453, 1.0549] |
| Fig. S13B | 850 nm | GLS activity | Cohen’s *d* | 0.4939 | [-0.9380, 1.8871] |
| Fig. S14 | 625 nm | HK2 | Cohen’s *d* | -7.2150 | [-12.2641, -2.1881] |
| Fig. S15A | 625 nm | LD area | Cohen’s *d* | -0.6307 | [-2.0358, 0.8225] |
|  |  | CD_L_/CH_L_ | Cohen’s *d* | -0.2796 | [-1.6629, 1.1261] |
| Fig. S15B | 625 nm | LD area | Cohen’s *d* | -1.2332 | [-2.7379, 0.3512] |
|  |  | CD_L_/CH_L_ | Cohen’s *d* | 0.1438 | [-1.2502, 1.5262] |
| Fig. S16A | 625 nm | Glutamine uptake | Cohen’s *d* | 0.0087 | [-1.3775, 1.3943] |
| Fig. S16B | 625 nm | GLS activity | Cohen’s *d* | -0.6046 | [-2.0072, 0.8442] |

Note: Effect sizes were calculated using independent biological experiment-level values. Eta-squared (η²) was used for one-way ANOVA analyses, epsilon-squared (ε²) for Kruskal–Wallis analyses, and Cohen’s *d* for two-group comparisons. The 95% confidence intervals correspond to the reported effect-size estimates. For η² and ε², confidence-interval bounds generated by approximation were constrained to the theoretical range of 0 to 1 because these metrics are bounded measures. For Cohen’s *d*, the sign was retained to indicate the direction of the effect based on the comparison direction used in the corresponding analysis.
